# Reliability analysis of backward erosion piping in an embankment dam considering the spatial variability of soil properties

**DOI:** 10.1038/s41598-025-09175-9

**Published:** 2025-07-04

**Authors:** Fengdong Chi, Claudio Carvajal, Pierre Breul, Laurent Peyras

**Affiliations:** 1 Aix-Marseille University, INRAE, RECOVER, Aix-en-Provence, 13182 France; 2 Université Clermont Auvergne, CNRS, Clermont Auvergne INP, Institut Pascal, Clermont-Ferrand, 63000 France

**Keywords:** Random fields, Initiation probability, Random finite element method, Reliability analysis, Backward erosion piping, Embankment dam, Natural hazards, Civil engineering

## Abstract

The spatial variability of soil seepage parameters is an essential component of the uncertainty of earth dams. This paper presents a method for backward erosion reliability analysis that considers the spatial variability of seepage parameters in earth dams to assess the distribution of the Factor of Safety (FoS) and the initiation probability (*P*_*f*_). In this study, the Karhunen-Loeve method is used to generate Random Fields (RFs) of soil hydraulic properties, and Monte Carlo simulations (MCS) are used to generate realisations of these RFs, which are then introduced into the numerical model to assess the spatial variability of the seepage analysis results. The hydraulic gradient distribution is compared with the critical hydraulic gradient to assess the FoS and the *P*_*f*_ due to backward erosion at each point of the dam. This study also presents the effect of water level *H*, permeability anisotropy coefficient *ξ*, and RFs parameters (coefficient of variation COV_Ks_, correlation length *L*_*h*_ & *L*_*v*_) on the FoS and *P*_*f*_ of Backward Erosion Piping (BEP). The results illustrate that the initiation probability *P*_*f*_ increases with *H*, COV_Ks_ and *ξ*. The initiation probability *P*_*f*_ is not sensitive to the vertical correlation length, but may decrease with the horizontal correlation length. Furthermore, the study discusses the potential initiation locations of internal erosion and presents results based on criteria using both local and global hydraulic gradients.

## Introduction

As one of the most common types of earth dam failure, internal erosion has been a major concern for experts and scholars worldwide. Statistics on the failures of large earth dams show that almost 50% of the failures are due to internal erosion, the rest are mainly attributed to overtopping^1^ and a small fraction to the sliding mechanism^2^. Traditional seepage analysis adopts the deterministic analysis method and takes hydraulic gradient or flow velocity as the evaluation criterion. However, there are significant uncertainties associated with the geotechnical parameters used in seepage analysis and in assessing the risk of internal erosion. It is interesting to use probabilistic methods to quantify these uncertainties and to assess the reliability of seepage analysis results^3,4^. Probabilistic methods provide a systematic approach to incorporating uncertainty into the analysis by considering the probability distribution of input parameters^5–8^. The variability and uncertainty associated with these parameters can be effectively captured by assigning probability distributions to geotechnical parameters such as permeability^9^, porosity^10^, and soil strength^11^. This approach allows for a more comprehensive understanding of the potential range of outcomes and the associated risks.

Backward erosion piping is one form of internal erosion that can cause the failure of an earth dam. Several scholars have studied backward erosion using analytic and deterministic analyses: Some studies^12–15^ have proposed models that are briefly introduced in ICOLD Bulletin 164^16^. Terzaghi’s model^12^ showed that backward erosion piping occurs when the effective stress at the downstream toe of the dam reaches zero. In this model, the critical hydraulic gradient can be estimated based on the soil’s specific gravity and porosity. Sellmeijer’s model^13^ was proposed based on visual observations of experiments of horizontal flow. In this model, the factor of safety (FoS) is influenced by the resistant factor *F*_*R*_, scale factor *F*_*S*_, and geometrical shape factor *F*_*G*_. Schmertmann^14^ proposed an analytical/empirical model based on Sellmeijer’s research work. This model uses an extensive set of experimental results from flume tests to estimate the safety factor against piping at any location along an anticipated progression path. Hoffmans^15^ described an analytical/empirical model based on Darcy’s law. The equation for predicting the critical hydraulic gradient comprises the critical hydraulic gradients of Shields (critical flow resistance in the pipes) and the critical hydraulic gradients of Darcy (seepage resistance in the aquifer). Fell et al.^17^ proposed a method to approximately estimate the time for the progression of internal erosion piping and the development of a breach leading to failure in embankment dams and their foundations. Benaissa et al.^18^ modeled water-clay mixtures flowing through a cylindrical pipe domain using the computational fluid dynamics (CFD) simulation method, and the results were validated by conducting a hole erosion test. The study showed that the fine particles are separated from the skeleton under the seepage flow, which reduces soil strength and increases porosity. El Shamy et al.^19^ investigated the interaction between eroded soil particles, water flow, and soil skeleton. They used Navier–Stokes equations to solve problems involving continuous fluids and employed particle flow software (PFC) to simulate fine particles with internal piping erosion. Guo & Yu^20^ coupled computational fluid dynamics and discrete element method (DEM) computational models, using CFD-DEM to simulate internal soil erosion and predict scouring depth and soil erosion rate. The study demonstrated the effectiveness of the particle flow software in calculating the degree of damage caused by internal erosion to the dam. Peng & Rice^21^ employed an inverse analysis to interpret pore pressure data and observations during backward erosion piping (BEP) initiation and progression in sandy soils, identifying critical hydraulic conditions for stages such as channel initiation and sand fluidization.

However, the soil properties of dams are associated with uncertainty, leading to uncertainty in the factor of safety against backward erosion piping. Some scholars have conducted reliability analysis to estimate the initiation probability of the dam. Most of these studies use the random variables (RVs) method, while several studies use the random fields (RFs) method that is capable of considering the spatial correlation characteristics of the geotechnical properties in the study area. Cho^22^ performed a reliability analysis of seepage through an embankment and its foundation to study the effects of uncertainty due to the spatial heterogeneity of permeability on seepage flow. Liu et al.^23^ investigated the influence of five commonly used autocorrelation functions (ACFs) on the seepage flow problem based on Cho’s model. The study found that the squared exponential (SQX) and single exponential (SNX) ACFs may overestimate and underestimate the seepage flow rate, respectively, while the differences between different ACFs are not significant. Tan et al.^24^ investigated the influence of the spatial variability of hydraulic parameters on the flow in earth dams by considering soil-water characteristic curve parameters as lognormal random fields. Liang et al.^25^ developed a numerical algorithm and conducted simulations to study backward erosion progressions in geologic media with spatially stochastic parameters, including void ratio, permeability, and the ratio of the particle contents. The study assessed the probability of failure due to seepage instability at the site investigated. Hekmatzadeh et al.^26^ studied the performance of four cutoff wall configurations and evaluated them stochastically using the random finite element method. The study found that the probabilities of failure for different cutoff wall configurations are similar when considering isotropic permeability, but there are noticeable differences in anisotropic situations. Moreover, Chi et al.^27^ compared the effects of three types of permeability RFs (stationary, conditional, and non-stationary RFs) on hydraulic parameters such as flow velocity, hydraulic gradient, pore water pressure, and flow rate in stochastic seepage analysis. The results showed that the different types of permeability RFs had a significant impact on flow velocity, a moderate impact on hydraulic gradient, and a minor impact on pore water pressure.

Although research on the reliability analysis of backward erosion is limited, the main objective of this study is to develop a probabilistic approach to evaluate the safety of earth dams by focusing on the backward erosion mechanism. The specific research objectives of this study are: (1) to build a hydraulic model for the probabilistic evaluation of a dam taking the internal erosion mechanism into account; (2) to analyse the spatial variability of internal flows and their impact on assessing the backward erosion mechanism in an earth dam; (3) to includes a parameter sensitivity analysis for the upstream water level and the spatial variability of permeability. The results of backward erosion are discussed and compared using the local hydraulic gradient method (Terzaghi method) and the global critical gradient method (Sellmeijer method & Hoffmans method). The results of the position of initiation of backward erosion are discussed and compared, based on the FoS and initiation probability *P*_*f*_ assessed at each point of the dam.

The originality of this study does not lie in the development of new erosion models or novel mechanistic descriptions of backward erosion. Rather, it stems from the integration of existing backward erosion models (e.g., Terzaghi, Sellmeijer, and Hoffmans criteria) with a probabilistic framework that accounts for the spatial variability of soil properties through random fields. This approach enables a spatially-resolved assessment of the factor of safety (FoS) distribution (mean and standard deviation) and the initiation probability of internal erosion at each point of the dam, thus providing valuable insights into dam safety under uncertainty.

BEP is a progressive, time-dependent process involving detachment, transport, and enlargement phases. It should also be clarified that the scope of this study is limited to the initiation phase of backward erosion, which represents a necessary condition for failure. The initiation criterion—based on the comparison between the local hydraulic gradient and the critical gradient—is commonly used to identify high-risk zones in geotechnical assessments^16^.

The article is structured as follows to achieve these goals. Section 2 briefly presents the methods used for the seepage analysis of earth dams and the backward erosion mechanism. Section 3 introduces the case of an earth dam, the simulation of random fields and the procedure of a finite element method combined with Monte Carlo simulations (MCS). Section 4 presents the results of the probabilistic seepage analysis, which provides the mean and standard deviation of FoS and initiation probability *P*_*f*_ for backward erosion piping initiation. Furthermore, global and parametric sensitivity analyses are conducted to investigate the impact of the uncertainty parameters of spatial variability on backward erosion. Section 5 discusses the initiation position of backward erosion and the initiation probability *P*_*f*_ obtained by using a local or global hydraulic gradient. Finally, Sect. 6 presents the main conclusions.

## Materials and methods for seepage analysis and backward erosion initiation analysis

### Seepage analysis

Internal erosion often causes damage to hydraulic structures and foundations. According to a survey conducted by Foster et al.^28^ on more than 11,000 large earth dams, 45% of failure modes are caused by internal erosion.

All dams have some seepage as the impounded water seeks paths of least resistance through the dam and its foundation. The seepage analysis model used in this study is based on governing equations solved by the finite element method. Richards’ equation^29^ is used to describe the flow through porous media. The generalised Darcy’s law (see Eq. (1)) and steady-state are assumed in this study. The governing equation for steady-state seepage flow is written as Eq. (2).

1 $$v = \frac{Q}{A} = k \cdot \frac{{h_{1} - h_{2} }}{L} = ki$$

2 $$\frac{\partial }{\partial x}\left( {k_{x} \frac{\partial h}{{\partial x}}} \right) + \frac{\partial }{\partial z}\left( {k_{Z} \frac{\partial h}{{\partial z}}} \right) = 0$$

Where *v* = flow velocity, *Q* = flow rate, *A* = cross-sectional area, *k* = permeability (hydraulic conductivity), *h*_*1*_-*h*_*2*_ = the elevation difference between the water levels, *L* = length of the soil sample, *i* = hydraulic gradient, *k*_*x*_ and *k*_*z*_ are the permeability of the soil along directions *x* and *z*, respectively, and *h* is the water level. The Optum-G2 software was used for the finite element analysis of seepage in this study.

This study considers a saturated-unsaturated seepage analysis in earth dams. Unsaturated seepage refers to the flow of water through the soil where the water content is less than the maximum capacity of the material. Compared with the saturated seepage calculation, the unsaturated seepage calculation requires considering the soil water retention curve (SWRC). The Van Genuchten model^30^ is considered in this study. The volumetric water content of the soil and permeability can be expressed as Eqs. (3 −1),  (3−2) :

3-1 $$S_{e} (\psi ) = \frac{{\theta - \theta_{r} }}{{\theta_{s} - \theta_{r} }} = \frac{1}{{\left[ {1 + \left( {\psi \alpha } \right)^{n} } \right]^{m} }}$$

3-2 $$K = K_{s} S_{e}^{1/2} \left[ {1 - \left( {1 - S_{e}^{1/m} } \right)^{m} } \right]^{2}$$

where: *S*_*e*_ *(ψ)* is a function of the volumetric moisture content of the soil, *ψ* is the matric suction, *S*_*e*_ is for the effective saturation, *θ* is the volumetric water content, *θ*_*s*_ is the saturated volumetric water content, *θ*_*r*_ is the residual volumetric water content, arameter *α* represents the suction corresponding to the inflexion point when the SWRC changes from the saturated state to the unsaturated state, parameter *n* represents the slope at the inflexion point of the SWRC, reflecting the change of volumetric water content in the initial intake stage, parameter *m* represents the soil parameters corresponding to the residual moisture content of the soil, *m =* 1–1/*n*. *K* is the hydraulic conductivity function, and *K*_*s*_ is the saturated hydraulic conductivity.

### Backward erosion initiation

As one of the main mechanisms of internal erosion, backward erosion piping commences with the detachment of particles at the exit of leakage paths through or under the embankment. The erosion process begins at a free surface on the downstream side of the dam. Backward erosion piping may occur when the hydraulic gradient (*i*) exceeds the critical hydraulic gradient (*i*_*cr*_). Piping refers to the movement and loss of fine-grained soil in the pores formed by the coarse-grained skeleton. The increase of pores in the soil eventually leads to the formation of seepage channels inside the soil, resulting in soil damage^28^. Internal erosion is a progressive, time-dependent process involving detachment, transport, and enlargement phases. This study focuses on backward erosion initiation, a common simplification that enables efficient spatial analysis under hydraulic uncertainty. ICOLD Bulletin 164^16^ recommends several methods to estimate the local and global critical hydraulic gradient for assessing backward erosion initiation.

The calculation formula for the local critical hydraulic gradient, proposed by Terzaghi^31^ and based on the force balance of unit soil in the seepage field, is given by Eq. (4) where *n*_*p*_ is the porosity, and *G*_*s*_ is the specific gravity of the soil. Physically, *i*_*cr*_ reflects the local resistance of soil to the detachment of particles under seepage. Zones with higher porosity or lower particle density exhibit lower *i*_*cr*_, indicating weaker structural integrity and higher susceptibility to backward erosion. The factor of safety (FoS) against backward erosion piping is defined as the ratio of the critical hydraulic gradient *i*_*cr*_ to the hydraulic gradient *i*, as shown in Eq. (5).

4 $$i_{cr} = \left( {G_{S} - 1} \right)(1 - n_{p} )$$

5 $${\text{FoS}} = \frac{{i_{cr} }}{i} = \left\{ \begin{gathered} \ge 1,safe \hfill \\ < 1,failure \hfill \\ \end{gathered} \right.\,\;$$

Regarding the global critical hydraulic gradient, there are several methods used in practical engineering to evaluate dam safety, such as the Sellmeijer method^32^, and the Hoffmans method^33^.

In the Sellmeijer model, the critical hydraulic gradient is determined as a product of three contributions: resistance factor, scale factor and geometrical shape factor (as shown in Eq. (6)):

6 $$\left\{ {\begin{array}{*{20}c} \begin{gathered} \frac{{H_{c} }}{L} = \frac{1}{c} = F_{r} F_{s} F_{g} \hfill \\ F_{r} = \eta \left( {G_{s} - 1} \right)\tan \vartheta \hfill \\ \end{gathered} \\ {F_{s} = \frac{{d_{70} }}{{\sqrt[3]{\kappa L}}}} \\ {F_{G} = 0.91\left( \frac{D}{L} \right)^{{\frac{0.28}{{\left( \frac{D}{L} \right)^{2.8} - 1}} + 0.04}} } \\ {\kappa = \frac{\upsilon }{g}k} \\ \end{array} } \right.$$

where *H*_*c*_ (m) is the critical pressure head; *L* (m) is the seepage length (base length of the embankment); *H*_*c*_/*L* is the critical hydraulic gradient; *D* (m) is the thickness of the sand layer under the embankment; *c* is the erosion coefficient; *F*_*r*_ is the resistance factor; *F*_*S*_ is the scale factor; *F*_*G*_ is the geometrical shape factor; *d*_*70*_ (m) is the soil particle diameter for which 70% by weight of the soil is finer; *η* is the Whites drag coefficient; *ϑ* is the bedding angle of the sand; *κ* (m^2^) is the intrinsic permeability; *υ* (m^2^/s) is the kinematic viscosity, and *g* (m^2^/s) is the gravity.

In Hoffmans model, the most important parameters are permeability (*k*), particle size *d*_*50*_, *d*_*15*_ and the aquifer dimensions: the depth of the sand layer (*D*) and seepage length (*L*). The equations of Hoffmans backward erosion are expressed as Eq. (7):

7 $$\left\{ {\begin{array}{*{20}c} {\frac{{\left( {H_{1} - H_{2} } \right)_{c} }}{L} = \frac{{\sqrt g \left( {\psi_{lam,c} \left( {G_{s} - 1} \right)d_{15} } \right)^{3/2} }}{{\nu \sqrt {\alpha_{R} } }} + \left( {1 - \frac{{l_{c} }}{L}} \right)\frac{{d_{50} \nu }}{{l_{{\text{Re}}} kD}}} \\ {\frac{{l_{c} }}{L} = \exp \left( { - \left( {\frac{{\alpha_{f} D}}{L}} \right)^{2} \frac{{\sqrt g \left( {\psi_{lam,c} \left( {G_{s} - 1} \right)d_{15} } \right)^{3/2} }}{{\nu \sqrt {\alpha_{R} } }}} \right)} \\ {\psi_{lam,c} = 0.2\left( {D^{ * } } \right)^{ - 0.3} } \\ {D^{ * } = d_{50} \left( {\left( {G_{s} - 1} \right)g/\nu^{2} } \right)^{1/3} } \\ \end{array} } \right.$$

where *H*_*1*_ is the upstream water level; *H*_*2*_ is the water level at the soil surface (usually taken as 0 m); (*H*_*1*_-*H*_*2*_)_*c*_/*L* is the critical hydraulic gradient; *l*_*c*_ is the ratio of critical pipe length and seepage length; *l*_*Re*_ is the geometrical length scale; *α*_*f*_ is the geometrical groundwater coefficient; *α*_*R*_ is the geometrical pipe coefficient; *ψ*_*lam, c*_ is the critical Shields parameter.

For both the Sellmeijer and Hoffmans models in the global approach, the global FoS_*global*_ is equal to the ratio between the global critical hydraulic gradient *i*_*cr_global*_ and the global hydraulic gradient *i*_*global*_, see Eq. (8):

8 $${\text{FoS}}_{global} = \frac{{i_{cr\_global} }}{{i_{global} }} = \frac{{H_{c} /L}}{H/L} = \frac{{H_{c} }}{H}$$

## Methodology for reliability analysis of backward erosion

### Case study presentation

**Fig. 1:**
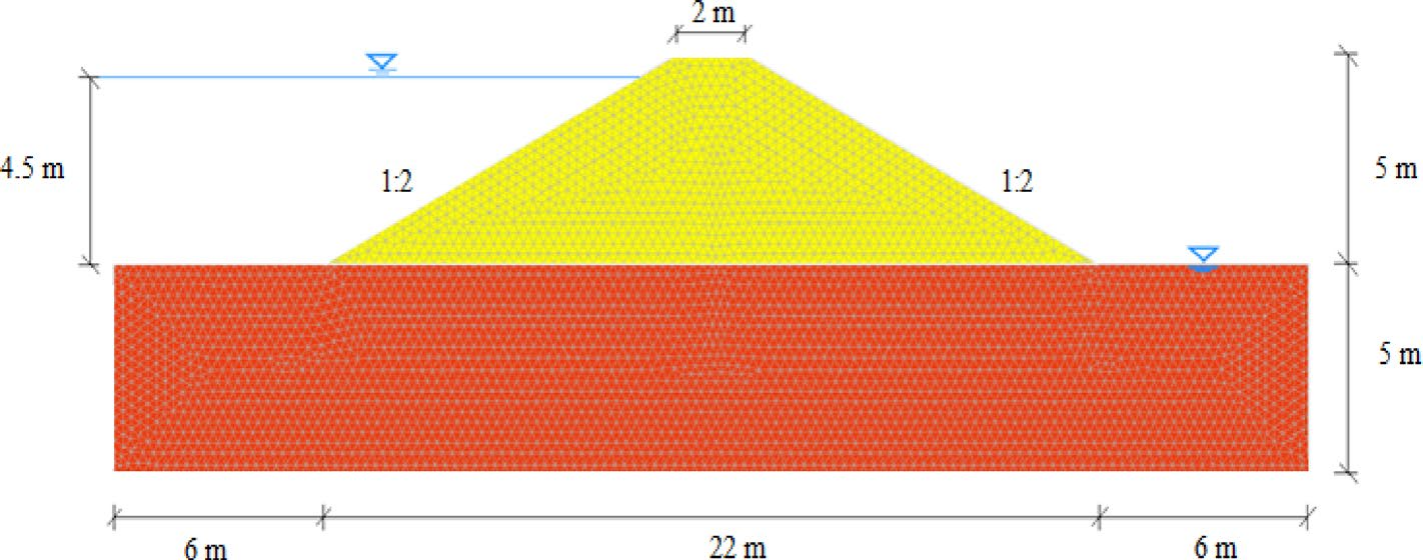
The finite element model in Optum G2.

In this study, the numerical modelling for the seepage analysis was performed within a hypothetical earth dam^22^ and the geometry of the dam is shown in Fig. 1. The embankment dam is 5 m high, with upstream and downstream slopes of 2 h:1 v, and is placed on a 5 m thick soil foundation. This simplified geometry, commonly used in benchmark studies^23,34^, facilitates the analysis of spatial variability in soil hydraulic properties and allows comparison with previous studies. The predicted flow rate and hydraulic gradient are consistent with published results, supporting the validity of the modeling approach. The research focused on the seepage analysis and backward erosion of the dam under steady-state saturated-unsaturated flow conditions.

The studied domain was discretised to 8009 triangular elements with 4154 nodes. A mesh convergence analysis was conducted to verify that the output deterministic analysis results—particularly the factor of safety (FoS) and flow rate (*Q*) —stabilized with increasing mesh refinement. The results are presented in Table 1. |*ε*(*Q*)| represents the relative difference in flow rate between a given mesh and the next finer mesh, while |*ε*(FoS)| denotes the relative difference in the factor of safety. Both metrics are used to evaluate the sensitivity of the results to mesh refinement and to assess numerical convergence. As shown, beyond approximately 8000 elements, further refinement had a minimal effect on the estimated FoS and flow rate. In addition, the adopted mesh size of 0.25 m satisfies the criterion^35^, which recommends that the maximum element length should be less than half of the autocorrelation distance in any direction. Therefore, the mesh with 8009 triangular elements and 4154 nodes was selected for all the subsequent probabilistic simulations, balancing accuracy and computational cost.

**Table 1:** Mesh convergence analysis for flow rate and factor of safety (FoS).

Case	Elements	Nodes	Mesh size (m)	Flow rate Q (m³/day/m)	FoS	|ε(Q)|	|ε(FoS)|
1	2060	1106	0.5	1.487	1.773	2.04%	10.70%
2	4007	2110	0.38	1.518	1.601	0.98%	5.00%
3	6084	3171	0.33	1.533	1.525	0.65%	4.28%
4	8009	4154	0.25	1.543	1.462	0.52%	3.51%
5	10,123	5228	0.23	1.551	1.413	0.32%	3.11%
6	12,054	6210	0.22	1.556	1.370	–	–

The hydraulic parameters (*K*_*s*_, *θ*_*r*_, *θ*_*s*_, *α*, *n*) adopted for the embankment and foundation can be found in Cho’s study^22^, and are in turn derived from the soil database^36^. The soil parameters (specific gravity *G*_*s*_ and porosity *n*_*p*_) that contribute to the resistance of backward erosion piping based on Terzaghi criteria (critical gradient *i*_*cr*_) can be found in Mainguenaud’s study^37^ (see Table 2). These parameters are representative of moderately well-graded sandy soils. They are consistent with laboratory-tested materials from a real French dam site^38^, and their classification aligns with the Unified Soil Classification System^39^.

Moreover, the seepage analysis results—such as flow rate (see Table 1) and hydraulic gradient (see Fig. 9a)—are in close agreement with those reported in benchmark studies^22,23^.

**Fig. 2:**
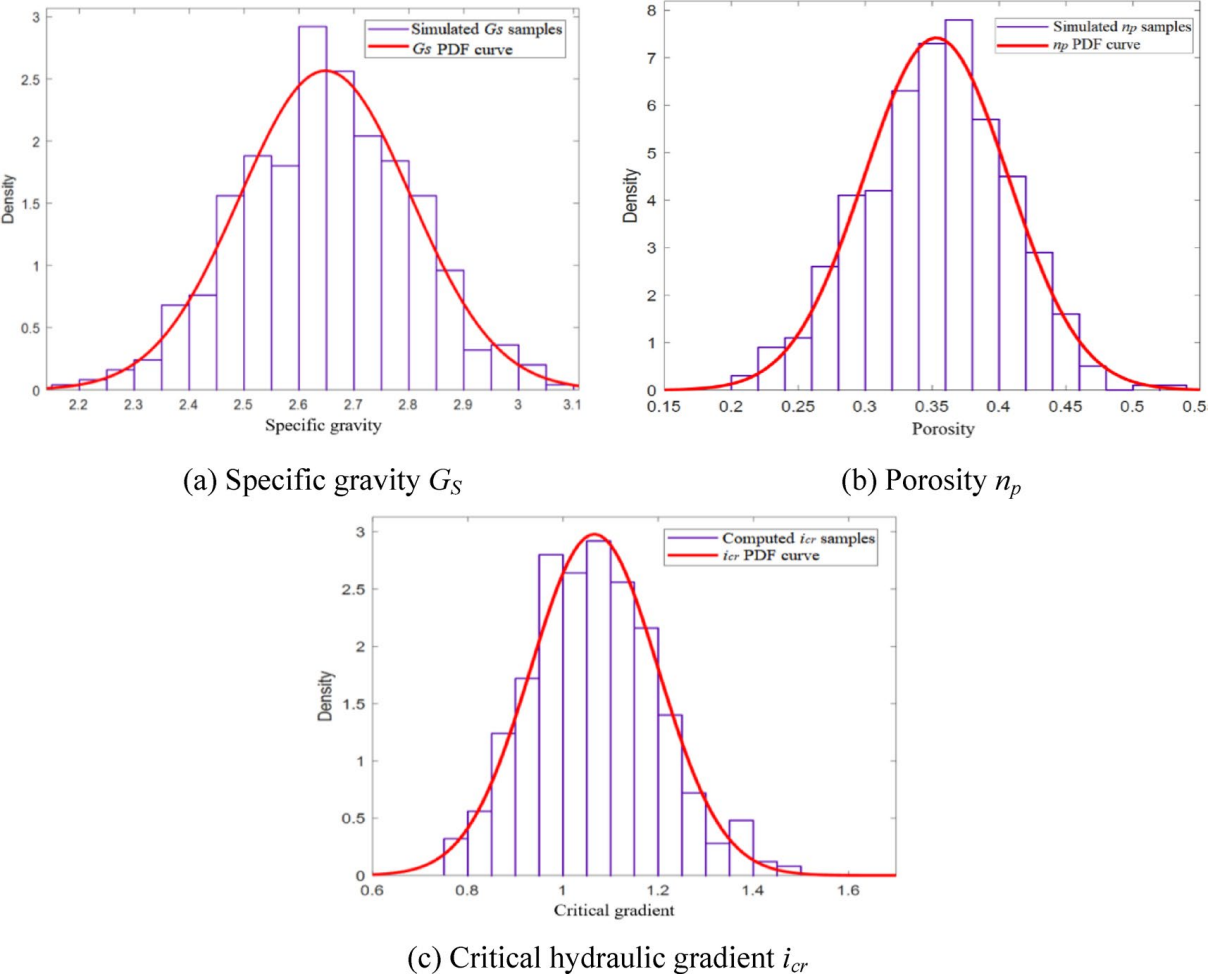
Distribution of soil parameters about backward erosion strength.

By simulating random samples of *G*_*s*_ and *n*_*p*_ according to the probability distribution shown in Table 2, it is possible to use Eq. (4) to calculate the critical gradient *i*_*cr*_ for each sample of *G*_*s*_ and *n*_*p*_. The resulting sample of critical gradient values can be used to estimate the mean and standard deviation of the critical gradient. Figure 2a,b show the histograms and probability density function (PDF) curves for the simulated samples of *G*_*s*_ and *n*_*p*_ respectively. Figure 2c shows the histogram and the fitted PDF curve for samples of calculated critical gradient values.

**Table 2:** Statistical properties of soil parameters used for backward erosion analysis.

Parameter	Mean	COV	Auto-correlation distance
*K*_*s*_	0.864 m/day (10^− 5^ m/s)	0.75	*L*_*h*_=20 m, *L*_*v*_=2 m
Critical hydraulic gradient *i*_*cr*_	1.06	0.255	
Specific gravity *G*_*s*_	2.65	0.06	
Porosity *n*_*p*_	0.357	0.055	
*θ*_*r*_	0.045	–	–
*θ*_*s*_	0.430	–	–
*α* (m^− 1^)	1.478	–	–
*n*	2.68	–	–
Anisotropy coefficient *ξ* (*K*_*h*_/*K*_*v*_)	1	–	–

**Fig. 3:**
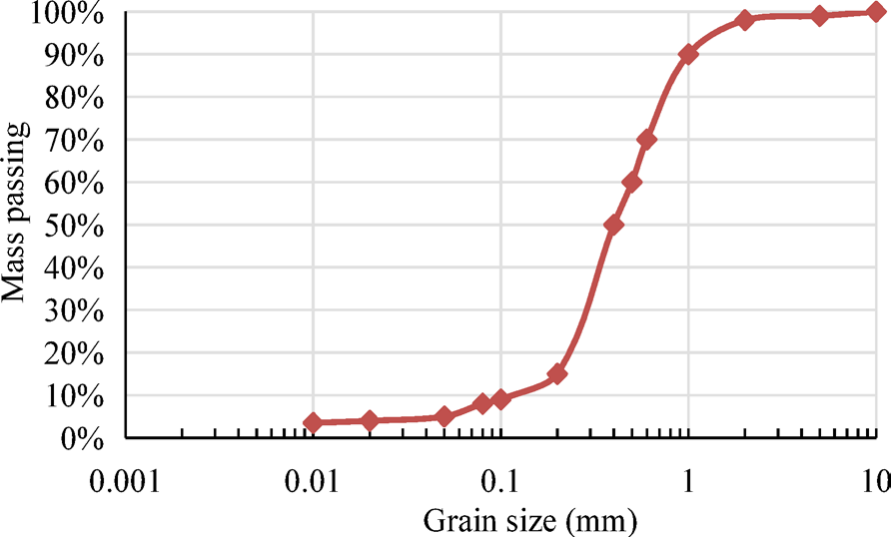
Grain size distribution for dam.

**Fig. 4:**
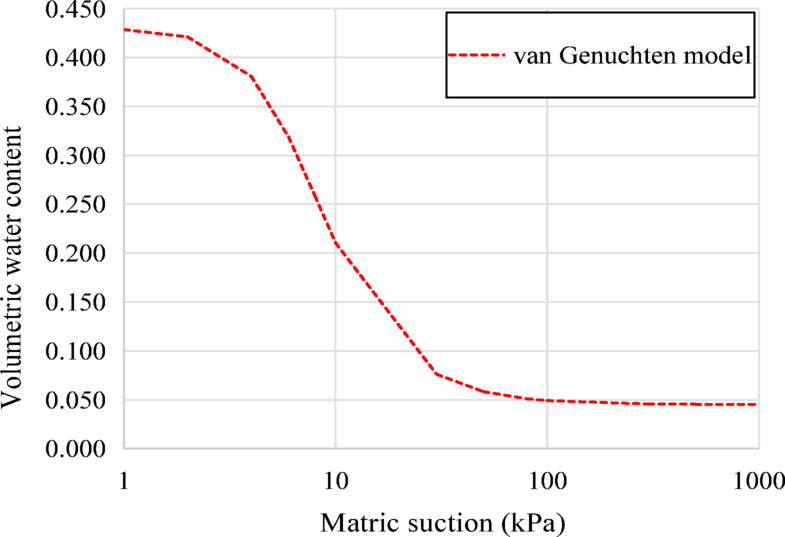
SWRC curves of van Genuchten model.

Figure 3 presents the grain size distribution curves used to determine the particle sizes *d*_*70*_, *d*_*50*_, and *d*_*15*_. Figure 4 shows the soil-water retention curve used in this study, which is based on the Van Genuchten model fitting parameters used in Cho’s study^22^. The prescribed boundary conditions for this model are as follows: the upstream water level *H* is assumed to be 9.5 m, and the downstream water table coincides with the surface of the dam foundation.

In this research, the Auto-Correlation Function (ACF) method, more specifically the single exponential autocorrelation function (SNX), was employed to generate random fields and represent the spatial variability of soil property parameters. The correlation length of each ACF type is associated with its Scale of Fluctuation. Regarding SNX, the Scale of Fluctuation used in this investigation is twice the correlation length. The values for *L*_*h*_ (horizontal correlation length) and *L*_*v*_ (vertical correlation length) were selected as 20 m and 2 m, respectively, based on assumptions made in earlier research^24,40,41^.

As mentioned in Sect. 2.2, besides the Terzaghi criterion based on the local hydraulic gradient, simplified methods based on the global hydraulic gradient (such as the Sellmeijer and Hoffmans methods) can also be used to assess the risk of the backward erosion of an earth dam. Compared to the Terzaghi method, which considers variable parameters such as permeability *K*_*s*_, specific gravity *G*_*s*_, and porosity *n*_*p*_, as shown in Table 2, the Sellmeijer and Hoffmans methods require considering additional variable parameters, as illustrated in Table 3.

**Table 3:** The statistical properties of additional parameters used in the Sellmeijer method & Hoffmans’ method.

Parameters	Mean	COV	Comment
tan*ϑ*	0.676	0.04	Normal distribution, used in Sellmeijer method
*d*_*70*_ (10^− 3^ m)	0.6	1.5	Lognormal Distribution, used in Sellmeijer method
*d*_*50*_ (10^− 3^ m)	0.4	1.5	Lognormal Distribution, used in Hoffmans’ method
*d*_*15*_ (10^− 3^ m)	0.2	1.5	Lognormal Distribution, used in Hoffmans’ method
*η*	0.25	–	Used in Sellmeijer method
*υ* (m^2^/s)	1.0*10^− 6^	–	Used in Sellmeijer method
*α*_*R*_	6	–	Used in Hoffmans’ method
*α*_*f*_	5	–	Used in Hoffmans’ method
*D* (m)	5	–	Used in Sellmeijer & Hoffmans’ method
*L* (m)	22	–	Used in Sellmeijer & Hoffmans’ method
*g* (m/s^2^)	9.81	–	Used in Sellmeijer & Hoffmans’ method

The spatial variability of the bedding angle of sand and the soil particle diameters (e.g. *d*_*70*_, *d*_*50*_, and *d*_*15*_) are considered in this study, the suggested parameter ranges are based on some researchers’ works^32,33,42^. Other parameters can be considered constants due to the inherent properties of the earth dam and its operating environment. For example, when the water temperature is at 20 degrees Celsius, the kinematic viscosity and drag coefficient are identified (*υ* = 1.0*10^− 6^ m/s, *η* = 0.25). The comparison and discussion of these three methods in estimating the risk for backward erosion will be presented in Sect. 5.2.

### Simulation of random fields

A random field (RF) can describe the spatial correlation of material properties in different locations and represent their heterogeneity characteristics^43^.

The Karhunen–Loève expansion (K-L) method was selected in this study to discretise random variables and generate random fields in order to simulate the spatial variability of soil properties. This method has been widely used in geotechnical problems such as slopes^44^, tunnels^45^, and foundations^46^. The Gaussian random field *H*_*i*_ *(x*,*θ)* can be expressed as Eq. (9):

9 $$H_{i} (x,\theta ) = \mu + \sum\limits_{{i = 1}}^{\infty } {\sigma \sqrt {\lambda _{i} } } \varphi _{i} (x_{{RF}} )\xi _{i} (\theta ) \approx \mu + \sum\limits_{{i = 1}}^{{N_{{KL}} }} {\sigma \sqrt {\lambda _{i} } } \varphi _{i} (x_{{RF}} )\xi _{i} (\theta )\,\,\,\,\,(x \in \Omega )$$

where *µ* is the mean of the parameters in RFs, *σ* is the standard deviation of the parameters in RFs, *λ*_*i*_ and *ϕ*_*i*_(x_RF_) are the eigenvalues and eigenfunctions of the autocovariance function for the RF, respectively.

The truncation number *N*_*KL*_ is determined by evaluating the truncated error with a target threshold^47^. The first task is to determine the truncation term number *N*_*KL*_ once the necessary probabilistic parameters (mean, COV_Ks_, *L*_*h*,_ and *L*_*v*_) are defined. It can be achieved by evaluating the truncation error of a K-L expansion RF evaluated by Eq. (10) with a target accuracy. In this paper, the *N*_*KL*_ is determined for a *ε*_*KL*_ lower than 2%. As shown in Eq. (10), *ε*_*KL*_ is the variance-based error globally estimated in the RF domain Ω, x_RF_ is the coordinate of an arbitrary point in the field.

10 $$\varepsilon_{KL} = \frac{1}{\Omega }\int_{\Omega } {\left[ {1 - \sum\limits_{i = 1}^{{N_{KL} }} {\lambda_{i} \varphi_{i}^{2} \left( {{\mathbf{x}}_{{{\mathbf{RF}}}} } \right)} } \right]} d\Omega$$

### Methodology based on the stochastic finite element method

The Monte Carlo simulation method, also known as the stochastic simulation or random experiment method, is a numerical technique that uses repeated random sampling to obtain numerical results^48^. Monte Carlo simulation has been widely used as a reliability method to evaluate structural safety in geotechnical engineering^49–52^. Among the many methods of reliability analysis, it is a relatively accurate method provided that a sufficiently large number of simulations are carried out. The aim of this paper is to study the influence of the spatial variability of soil parameters on the reliability or initiation probability, which has to be resolved through probabilistic seepage analysis. This study uses the Monte Carlo simulation method to simulate the distribution of the factor of safety (FoS) to initiate backward erosion.

**Fig. 5:**
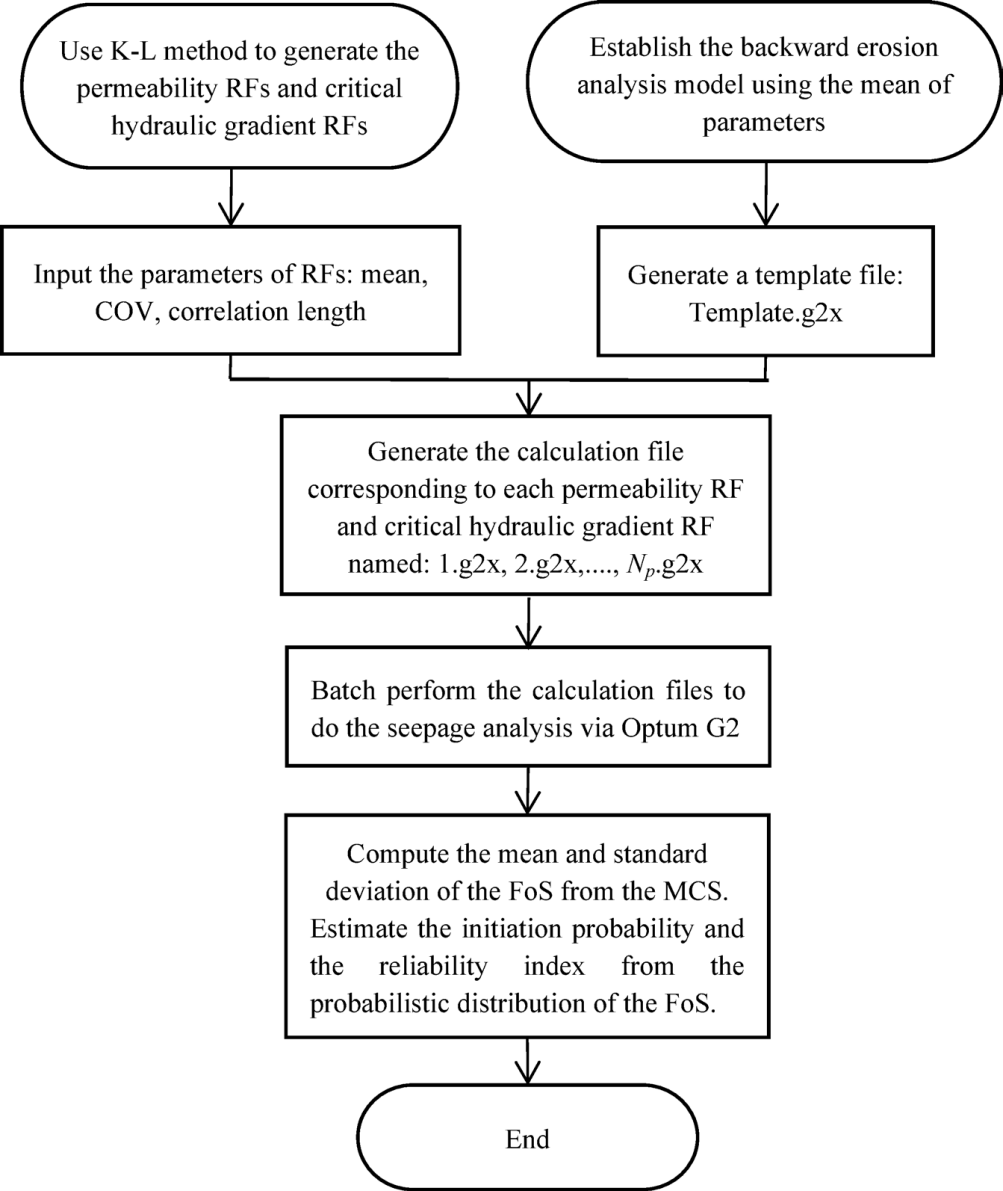
Flowchart of the reliability analysis.

This section presents the seepage analysis procedure using a random finite element method, as illustrated in Fig. 5. The procedure consists of the following main steps:

1. Identify the parameters with spatial variability and determine their statistical characteristics, including means, coefficients of variation (COVs) and distributions related to the seepage analysis. Then, select an appropriate autocorrelation function and the autocorrelation distances for the 2D random-field model.
2. Apply the Optum-G2 software to build the finite element analysis model for seepage analysis, using the mean values of input parameters. Then, obtain the coordinates (*x*_*i*_,*y*_*i*_) of the nodes, where i = 1, 2, …, *N*_*n*_, *N*_*n*_ being the number of the nodes in the finite element model. The deterministic seepage analysis model files are saved as the template file named ‘Template.g2x’. This file contains all the information used by OPTUM-G2, and it can also be accessed and viewed directly using a text editor.
3. Create a standard normal sample matrix using Latin hypercube sampling points. Next, apply the K-L expansion from Sect. 3.2 to simulate non-Gaussian random fields of spatially varying variables using the sample matrix and coordinates (*x*_*i*_,*y*_*i*_). Then, generate *N*_*p*_ realisations of non-Gaussian random fields that represent spatially varying permeability values in the physical space. Finally, assign each realisation to corresponding nodes in the finite element model. This stratified sampling technique improves the efficiency and uniformity of sample distribution compared to standard Monte Carlo sampling, especially for moderate sample sizes.
4. Substitute the mean values of the corresponding uncertain input parameters in the ‘Template.g2x’ file (created in step 2) with each pair of spatially varying variables (e.g. *K*_*s*_ and *i*_*cr*_ in Table 2) in each realisation of random fields from step (3). This process will generate *N*_*p*_ distinct new ‘Template.g2x’ input files, named 1.g2x, 2.g2x, …, *N*_*p*_.g2x.
5. Execute OPTUM-G2 using each of the new input files, namely 1.g2x, 2.g2x, …, *N*_*p*_.g2x, which were generated in step (5) to conduct seepage analysis. This procedure will yield *N*_*n*_ * *N*_*p*_ distinct factors of safety for backward erosion at each node of the finite element model across *N*_*p*_ realisations, resulting in a set of factors of safety denoted as Eq. (11). As shown in the matrix FoS: the elements in the 1st column present the FoS of all the nodes in the 1st seepage analysis realisation; the elements in the 1st row presen the FoS of the 1st node in all the seepage analysis realisations. For each realisation, the position of the dangerous (initiate backward erosion) node can be identified by looking for the minimum FoS in each column of matrix FoS. At each point of the dam (for each node *N*_*n*_), the mean and standard deviation of the FoS can be calculated from the elements in each row of matrix FoS following the *N*_*p*_ realisations from the MCS.

11 $$\:\mathbf{F}\mathbf{o}\mathbf{S}=\left\{\begin{array}{cccc}\text{Fo}{\text{S}}_{\text{1,1}}&\:\text{Fo}{\text{S}}_{\text{1,2}}&\:\cdots\:&\:\text{Fo}{\text{S}}_{1,{N}_{p}}\\\:\text{Fo}{\text{S}}_{\text{2,1}}&\:\text{Fo}{\text{S}}_{\text{2,2}}&\:\cdots\:&\:\text{Fo}{\text{S}}_{2,{N}_{p}}\\\:\cdots\:&\:\cdots\:&\:\cdots\:&\:\cdots\:\\\:\text{Fo}{\text{S}}_{{N}_{n},1}&\:\text{Fo}{\text{S}}_{{N}_{n},2}&\:\cdots\:&\:\text{Fo}{\text{S}}_{{N}_{n},{N}_{p}}\end{array}\right\}$$

(6) Evaluate the reliability index and the initiation probability due to internal erosion on the basis of the probabilistic distribution of the FoS obtained by the MCS. With MCS, the number of simulations must be sufficiently large to obtain the convergence of the variables of interest. The initiation probability can be defined directly as the ratio between the number of BEP initiations and the total number of simulations, which requires a very high number of simulations if the initiation probability is low. In order to limit the number of simulations, this study considered as variables of interest the mean and standard deviation of the FoS whose estimation converges more quickly. By adopting a probability distribution of the FoS with the mean and standard deviation values obtained from the MCS, the initiation probability *P*_*f*_ can be evaluated directly from the FoS distribution as the probability that the FoS is less than 1.

Although the hydraulic models used are standard, the stochastic integration—random field generation, batch simulation, and statistical post-processing—was fully customized. The contribution of this study lies in applying established physical models within a novel spatially probabilistic framework.

## Reliability analysis and results

This section presents a probabilistic seepage analysis based on Monte Carlo simulations using the random finite element model. The Terzaghi criterion was used to calculate the critical hydraulic gradient for the seepage failure of the dam. The location of the dangerous node with the lowest factor of safety (FoS), which is more prone to initiate backward erosion, could be identified. Then, the contours of *P*_*f*_ (initiation probability) were displayed to reflect the risk of backward erosion at each node directly, achieving the goal of data visualisation. Furthermore, a sensitivity analysis was performed to evaluate the significance of each stochastic input parameter on the FoS and the *P*_*f*_. The sensitivity analysis of FoS and *P*_*f*_ considered hydraulic parameters (Upstream water level *H* and Anisotropy coefficient of permeability *ξ* ) as well as random field parameters (COV_Ks_ and correlation length *L*_*h*_ & *L*_*v*_).

### Results of the reliability analysis of backward erosion

#### FoS distribution and initiation probability *P*_*f*_

**Fig. 6:**
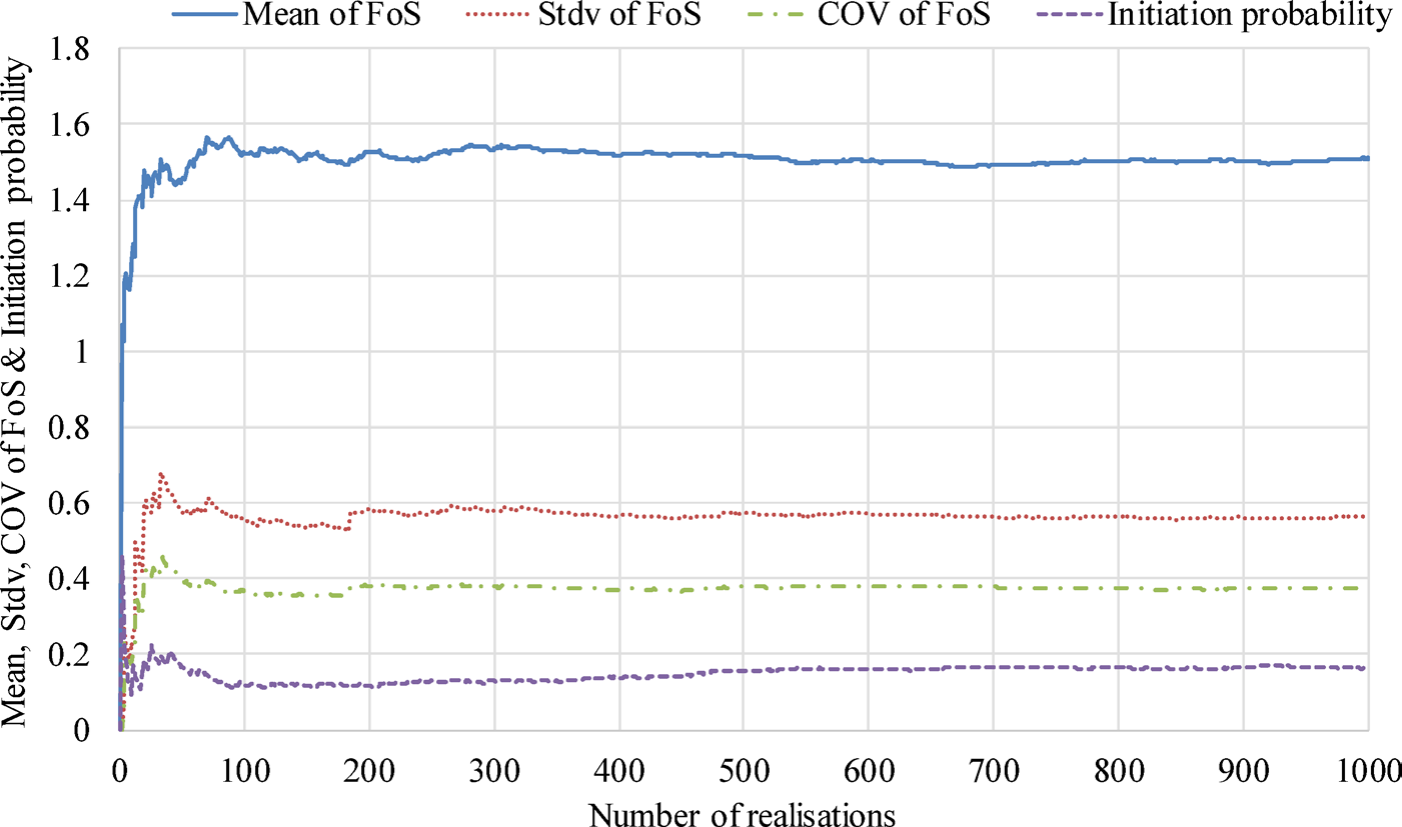
Convergence of the estimated mean, standard deviation, coefficient of variation of the FoS and initiation probablity.

The convergence analysis of the FoS of the node at the embankment toe was conducted through 1000 hydraulic simulations that incorporated the RFs (see Fig. 6). The estimated mean and standard deviation of the factor of safety (FoS) stabilize as the number of realizations exceeds 300, while the initiation probability *P*_*f*_ converges with more than 500 realizations. Given this convergence behavior, and considering that the estimated failure probability *P*_*f*_ is approximately 16.3% (see Figs. 6 and 11), 500 realizations (*N*_*p*_ = 500, as referenced in Sect. 3.3) were selected for each probabilistic seepage analysis. At this level, the standard deviation of the Monte Carlo estimate of *P*_*f*_ is approximately 1.65%, resulting in a 95% confidence interval of ± 3.3%, which is considered acceptable for engineering applications.

As done in previous studies, the permeability and critical hydraulic gradient are modeled by means of RFs to consider the spatial variability. The soil permeability of the earth dam has been considered a spatially random field that follows a lognormal distribution with a prescribed mean, variance, and spatial correlation structure^53^. Similarly, the critical hydraulic gradient has been modeled as RFs that follow a lognormal distribution^54^.

**Fig. 7:**
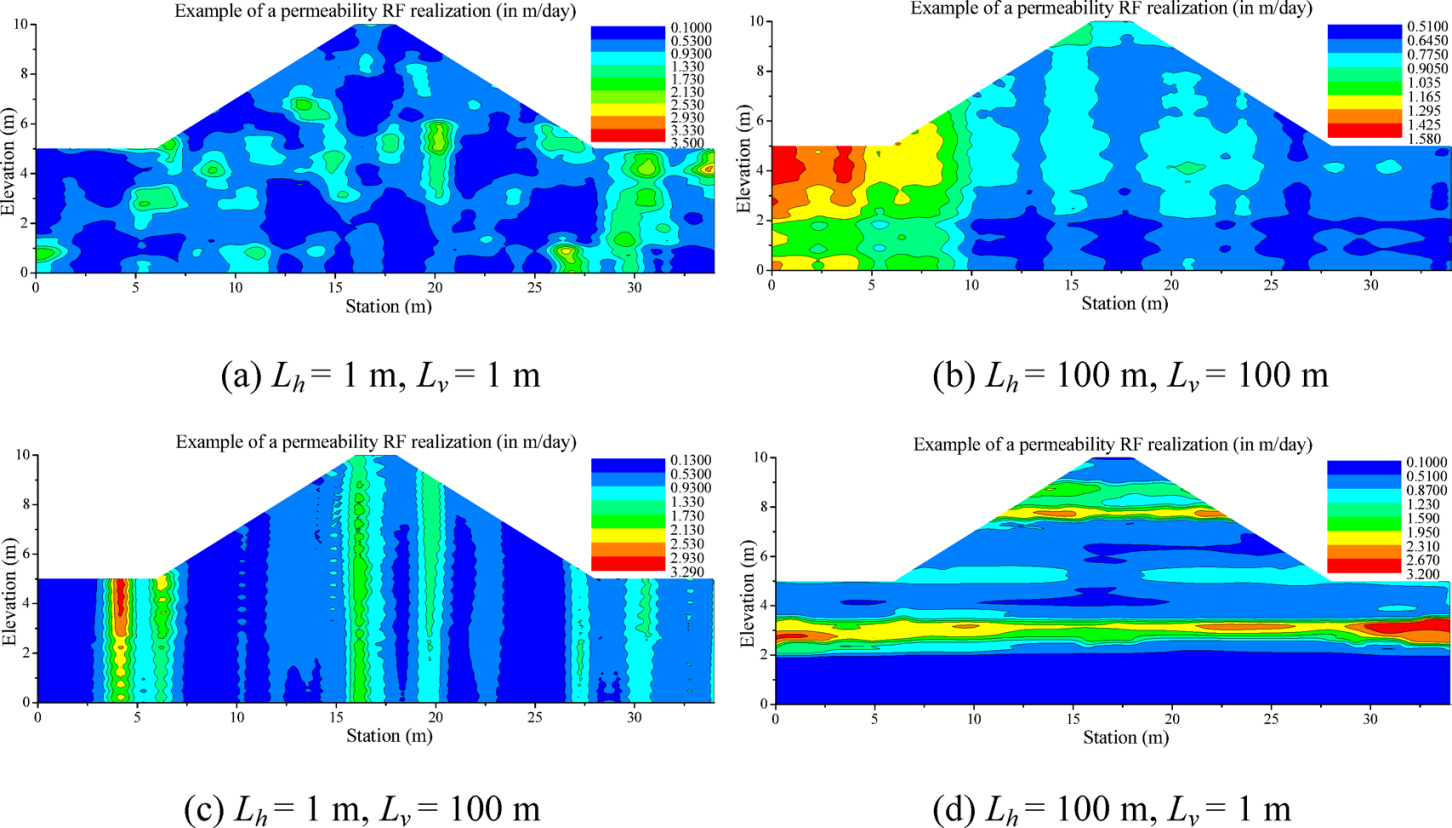
Spatial variability of permeability for the case using COV_Ks_=0.75.

Figure 7 shows the spatial distribution of permeability based on the same COV_Ks_ = 0.75 but with different correlation lengths used to generate RFs. A comparison between Fig. 7a,b reveals that the RFs with a higher correlation length correspond to a more homogeneous permeability RF. By comparing Fig. 7a,c (or Fig. 7d), it can be observed that the permeability distribution should follow the main direction of the correlation length. For instance, when the horizontal correlation length is significantly larger than the vertical correlation length, the contour of the permeability field will present a horizontal strip-like distribution, and vice versa.

**Fig. 8:**
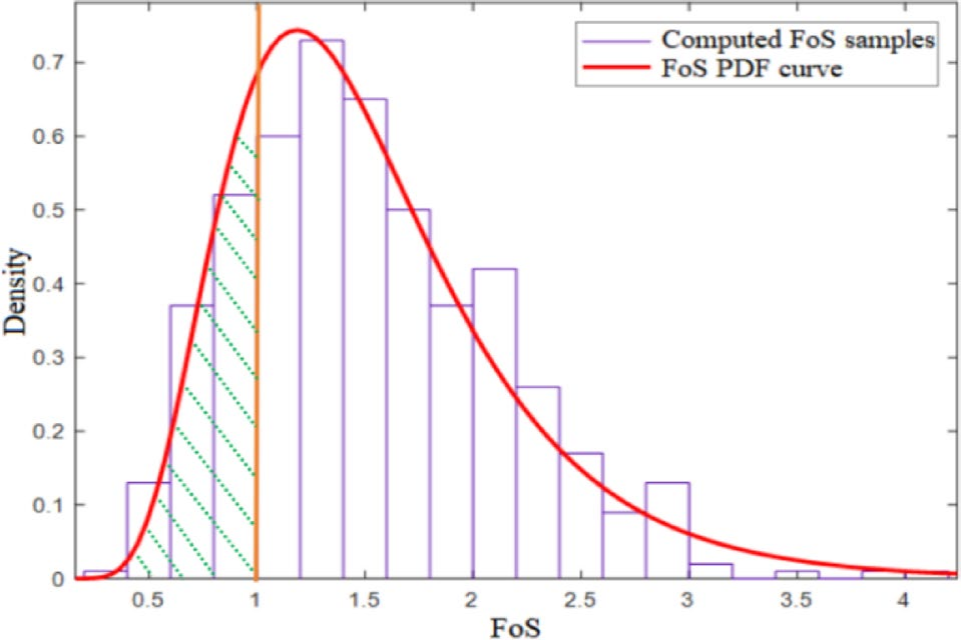
PDF of FoS against backward erosion obtained from the lognormal distribution of the input parameters.

**Fig. 9:**
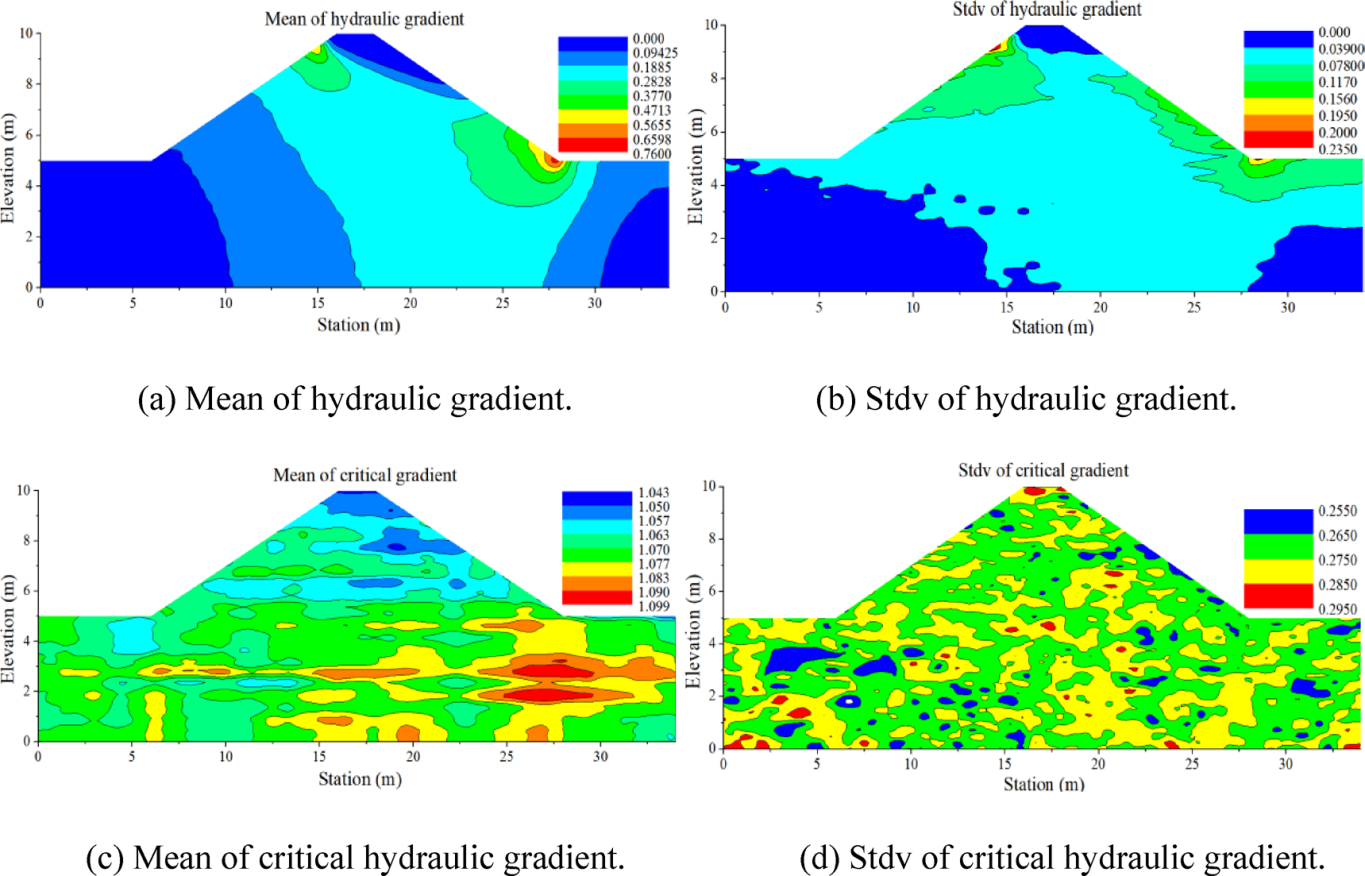
Spatial variability of the hydraulic gradient and critical hydraulic gradient from Monte-Carlo simulations.

**Fig. 10:**
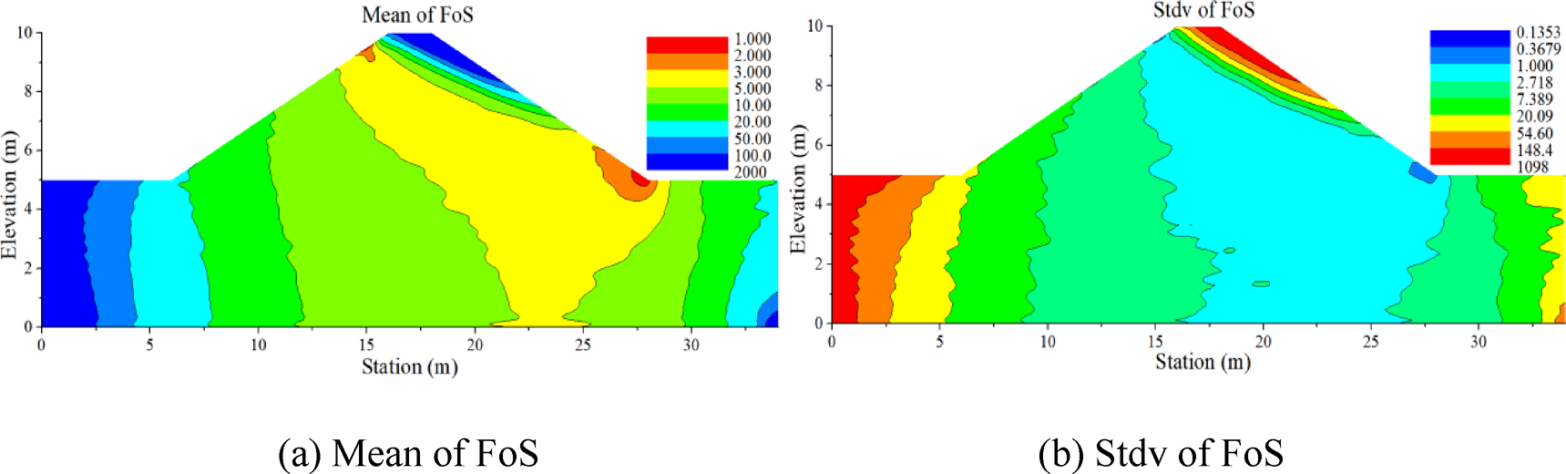
Spatial variability of the factor of safety FoS from Monte-Carlo simulations.

Figure 8 shows the probability density function of FoS against backward erosion for the configuration with COV_Ks_ = 0.75 and *H* = 9.5 m. The initiation probability *P*_*f*_ can be estimated from the probability density function fitted to the sample of FoS (*P*_*f*_ = 16.3%, in this case). Figure 9 displays the contours of the estimated mean and the standard deviation of the hydraulic gradient and critical hydraulic gradient. The figures for FoS (Fig. 10) are generated by combining the contours of the hydraulic gradient and critical hydraulic gradient using Terzaghi criteria.

**Fig. 11:**
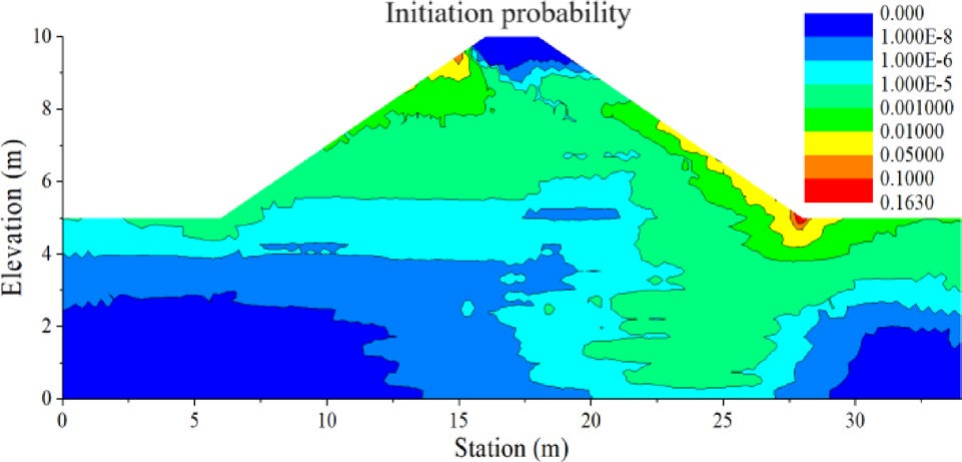
The contour of initiation probability of a dam in probabilistic backward erosion analysis.

The contours of *P*_*f*_ (Fig. 11) can be obtained by estimating the initiation probability from the FoS distribution for each node in the finite element model. The dangerous area in red with the highest backward erosion initiation probability *P*_*f*_ = 16.3% (also with the lowest estimated mean FoS = 1.48) is located at the toe of the embankment, as illustrated in Fig. 11. *P*_*f*_ can reach a high value due to the narrow geometry of the dam and the larger dispersion of input data (COV_Ks_, COV_Gs_, and COV_np_) used in this study. Among these factors, the narrow geometry of the dam is mainly reflected in the narrow width of the dam crest, steep slope, and low dam thickness, which can result in a higher hydraulic gradient. It is noteworthy that, although this study identifies a dominant critical zone near the dam toe, other scenarios may involve the alignment or connection of multiple marginally unstable regions. Such interactions could facilitate the formation of continuous erosion paths, particularly in highly heterogeneous soils.

#### Global sensitivity analysis

To assess the influence of key soil parameters (*K*_*s*_, *G*_*s*_, *n*_*p*_) quantitatively on the factor of safety (FoS) against backward erosion initiation, a global sensitivity analysis was performed using the Morris method. This method is widely used in engineering applications because it can effectively screen input variables, capture main effects and potential nonlinear interactions, and has relatively low computational costs^55^.

In this study, the Morris method was applied to three key input variables: saturated permeability (*K*_*s*_), specific gravity (*G*_*s*_), and porosity (*n*_*p*_), all of which play a direct role in the calculation of hydraulic gradient and critical gradient (*i*_*cr*_) based on the Terzaghi criterion (see Sect. 2.2). A total of 5,000 Monte Carlo simulations were used to evaluate the model response in terms of FoS.

**Fig. 12:**
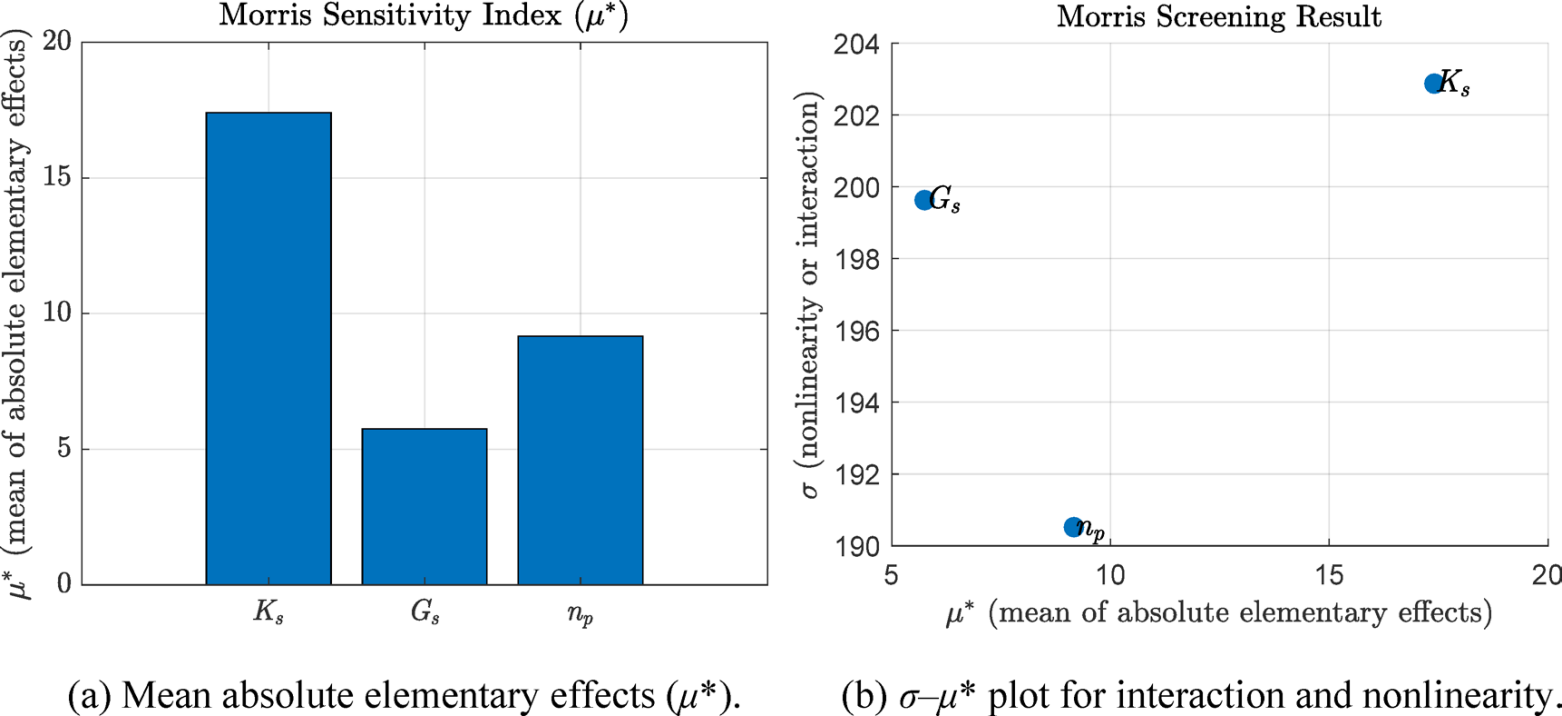
Morris global sensitivity analysis results (*K*_*s*_, *G*_*s*_ & *n*_*p*_).

Two indices were computed for each input parameter: the mean of the absolute elementary effects (µ*), which indicates the overall importance of the parameter, and the standard deviation (*σ*) of these effects, which reflects nonlinearity or interaction effects. The results are shown in Fig. 12a,b.

Figure 12a shows that *K*_*s*_ is the most influential parameter (µ* ≈ 17), followed by *n*_*p*_ (µ* ≈ 9) and *G*_*s*_ (µ* ≈ 6). Figure 12b illustrates the *σ*–µ* scatter plot, where all three parameters exhibit very high *σ* values (> 190), indicating significant nonlinearity or interaction effects. In particular, *K*_*s*_ not only dominates in terms of direct influence but also contributes to strong interactions with other variables. The results suggest that variations in *K*_*s*_ largely control the variability of FoS in the model, while *G*_*s*_ & *n*_*p*_ still participate in nonlinear behaviors under uncertain conditions, although with less impact.

These findings provide quantitative evidence supporting the prioritization of *K*_*s*_ in both uncertainty characterization and parameter calibration efforts. Accordingly, the following parametric sensitivity analysis in Sect. 4.2 focuses on the spatial variability of permeability (*K*_*s*_), including its coefficient of variation (COV_Ks_) and correlation lengths (*L*_*h*_ & *L*_*v*_), which were shown to have the most significant influence on the probabilistic assessment of backward erosion initiation.

### Parametric sensitivity analysis

Based on the study in Sect. 4.1.1, the dangerous nodes with the lowest FoS and highest *P*_*f*_ are generally located at the downstream toe of the embankment, where they readily initiate backward erosion. A sensitivity analysis was performed in this study to examine the impact of each stochastic input parameter on the FoS and the *P*_*f*_ of backward erosion. The value of FoS and initiation probability are mainly influenced by hydraulic parameters (water level *H* and anisotropy coefficient of permeability *ξ* ) as well as random field parameters (COV_K*s*_ and correlation length *L*_*h*_ & *L*_*v*_). The parameter values used in Sect. 4.1.1 (*H* = 9.5 m, *ξ* = 1, COV_Ks_ = 0.75, *L*_*h*_ = 20 m & *L*_*v*_ = 2 m) are considered as reference values. The sensitivity analysis considers different configurations of *H*, *ξ*, COV_Ks_, *L*_*h*_ & *L*_*v*_. Each configuration changes only one value while keeping the others at their reference values. To discuss the influence of these parameters on the safety of the dam, this section is divided into two parts. The first part investigates the effect of water level *H* and the coefficient of variation of permeability COV_Ks_ (Sect. 4.2.1), while the second part examines the influence of anisotropy coefficient *ξ* and correlation lengths *L*_*h*_ & *L*_*v*_ (Sect. 4.2.2). Thus, 20 configurations of water level and COV_Ks_ are included in Sect. 4.2.1, and presented in Table 4. Altogether, there are 28 independent configurations of anisotropy coefficient *ξ* and correlation lengths *L*_*h*_ & *L*_*v*_, (20 configurations in Table 4 and 8 configurations in Table 5). For each configuration, 500 groups of random field realisations were conducted, and the 500 corresponding simulations of seepage analysis were performed. It should be noted that the convergence of the backward erosion analysis results considering 500 realisations was validated previously (see Fig. 6).

#### Influence of water level *H* and coefficient of variation of permeability COV_Ks_

**Fig. 13:**
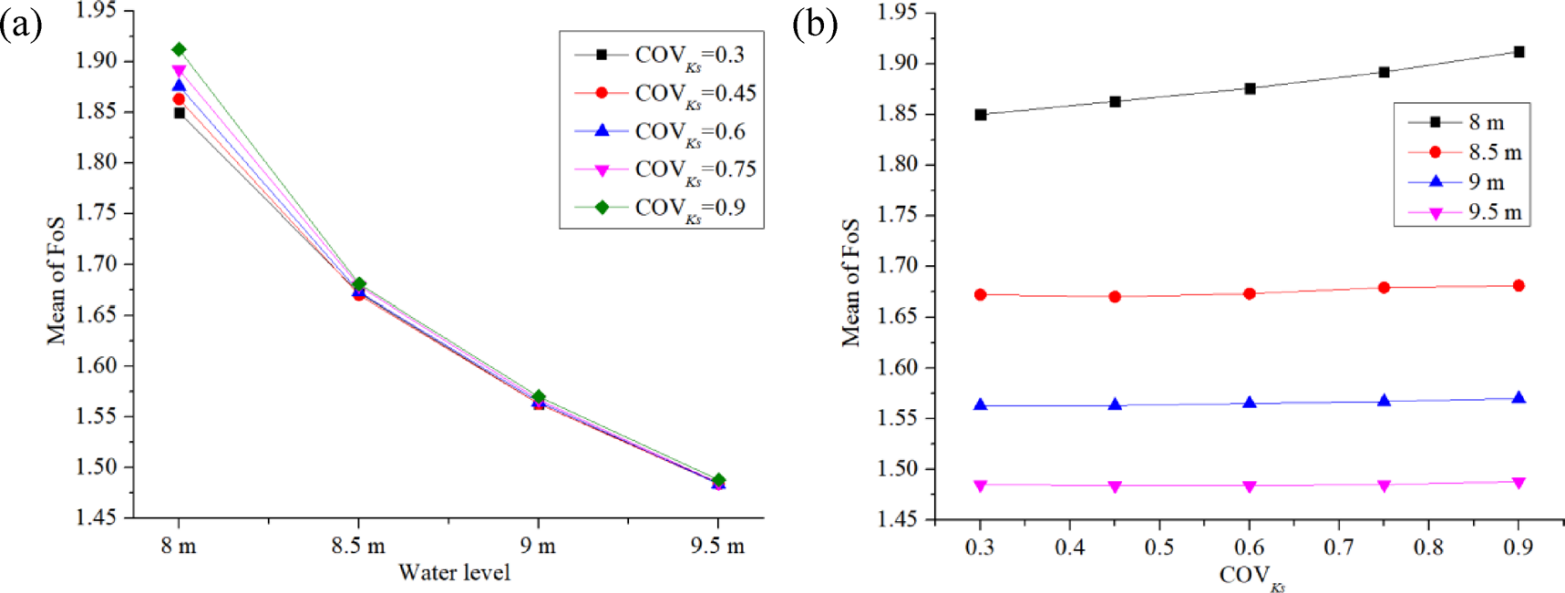
Influence of *H* and COV_Ks_ on *µ*_*FoS*_.

**Fig. 14:**
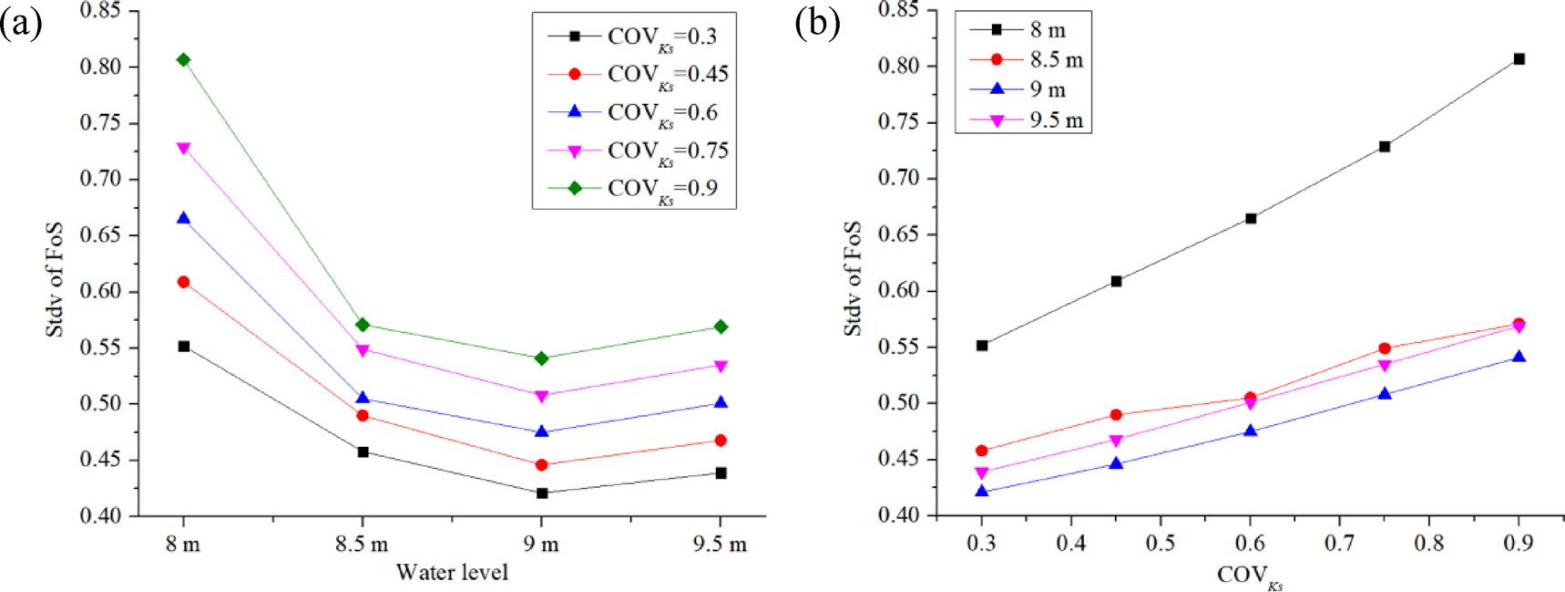
Influence of *H* and COV_Ks_ on *σ*_*FoS*_.

**Fig. 15:**
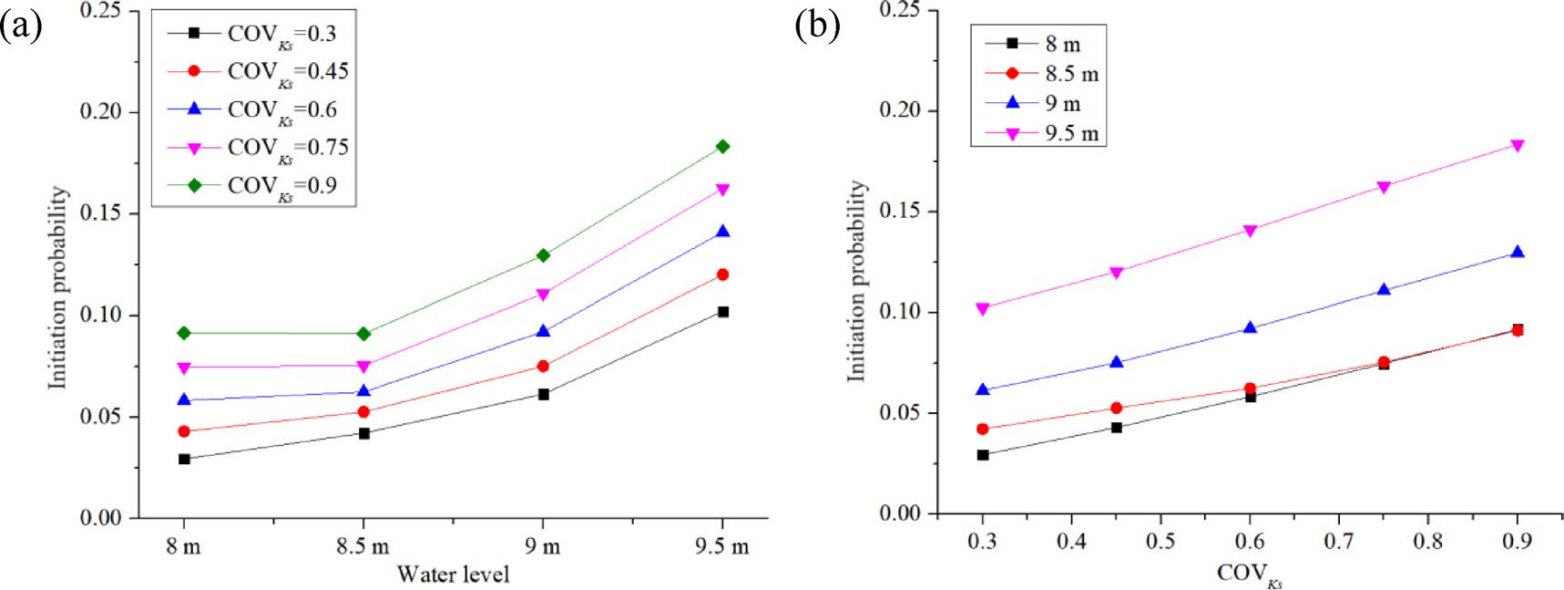
Influence of *H* and COV_Ks_ on *P*_*f*_.

**Fig. 16:**
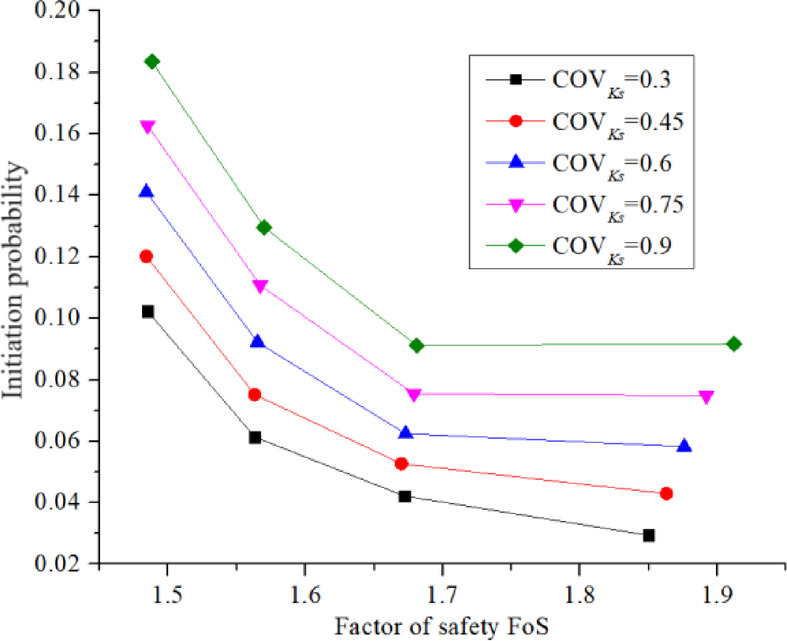
Influence of FoS and COV_Ks_ on *P*_*f*_.

This section examines the impact of water level *H* and COV_Ks_ on the FoS and *P*_*f*_ of backward erosion assessed at the downstream toe of the dam. All the cases in this section assumed a correlation length of *L*_*h*_ = 20 m and *L*_*v*_ = 2 m, and isotropic permeability (Anisotropy coefficient *ξ* = 1) for each node. Water level *H* and COV_Ks_ vary from case to case, as shown in Table 4. Figures 13 , 14, 15 illustrate the influence of *H* and COV_Ks_ on the estimated mean FoS (*µ*_*FoS*_), standard deviation of FoS (*σ*_*FoS*_), and initiation probability *P*_*f*_, respectively, which were obtained from the probabilistic seepage analysis. Figure 16 assists in explaining the results shown in Figs. 13 , 14, 15 illustrating the influence of *H* and COV_Ks_ on COV_FoS_ and the influence of FoS and COV_Ks_ on *P*_*f*_, respectively.

**Table 4:** The water level *H* and COV_Ks_ influence on backward erosion.

Parameter	Value
Upstream water level *H* (m)	8, 8.5, 9, 9.5
COV_Ks_	0.3, 0.45, 0.6, 0.75, 0.9
Anisotropy coefficient *ξ*	1
Correlation length *L*_*h*_ & *L*_*v*_ (m)	*L*_*h*_ = 20, *L*_*v*_ = 2

Figure 13a displays a significant decrease in the estimated mean FoS (*µ*_*FoS*_) with an increase in reservoir water level. The rate of decrease slows down as the water level increases from 8 m to 9.5 m, as shown in the curve graph. Figure 13b shows a slight increase in *µ*_*FoS*_ with the COV_Ks_ at a relative water level of 8 m, but no significant increase for the water level range of 8.5 m to 9.5 m.

Figure 14a,b show that the estimated standard deviation of FoS (*σ*_*FoS*_) decreases overall as the water level increases, assuming constant COV_Ks_. However, a slight increase in *σ*_*FoS*_ is observed when the water level rises from 9 m to 9.5 m. One possible explanation may be related to the position of the point considered in the evaluation of backward erosion (as discussed in Sect. 5.1).

Figure 15a illustrates that the rate of initiation probability *P*_*f*_ increment accelerates as the water level *H* increases. Figure 15b shows that the initiation probability *P*_*f*_ increases uniformly with COV_Ks_ (with a slightly lower rate of increase for the case with a water level of 8.5 m). In contrast, Fig. 16 shows that the initiation probability *P*_*f*_ decreases, and the rate of decrease slows down gradually with the increase in the factor of safety (FoS).

#### Influence of anisotropy coefficient *ξ* and correlation length *L*_*h*_ & *L*_*v*_

**Fig. 17:**
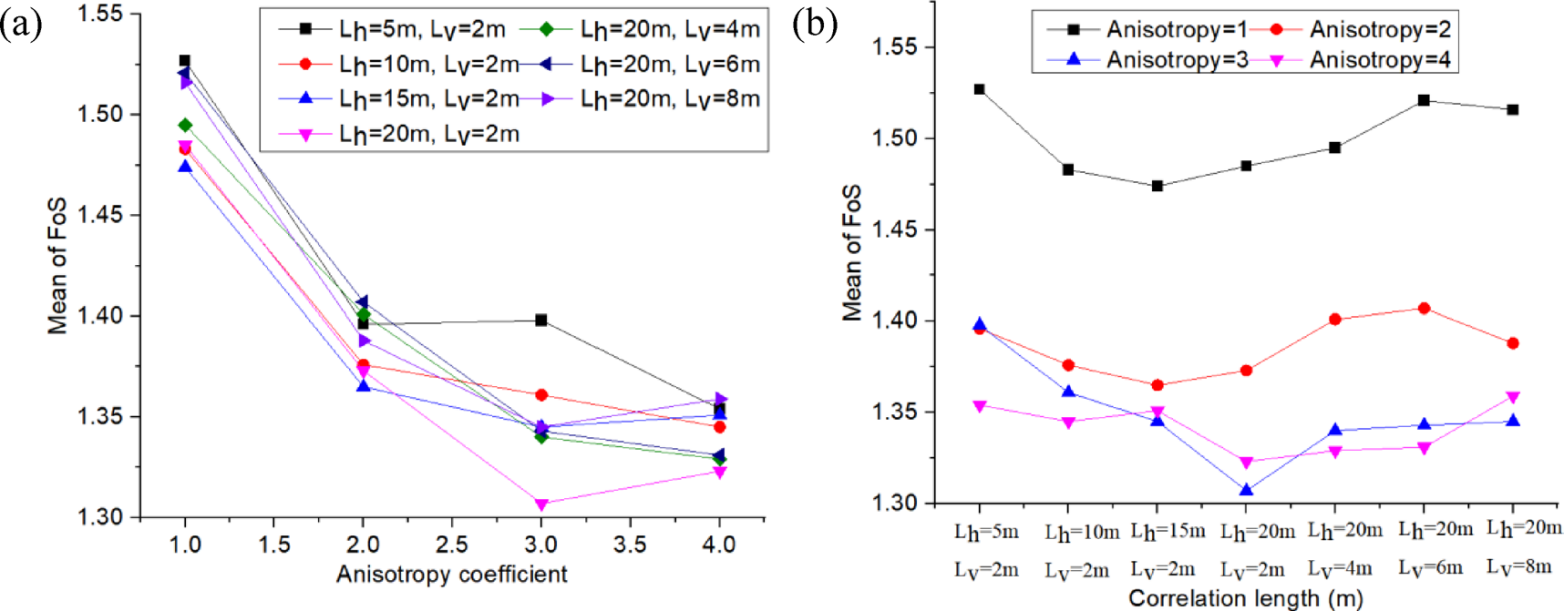
Influence of *ξ*, *L*_*h*_ and *L*_*v*_ on *µ*_*FoS*_.

**Fig. 18:**
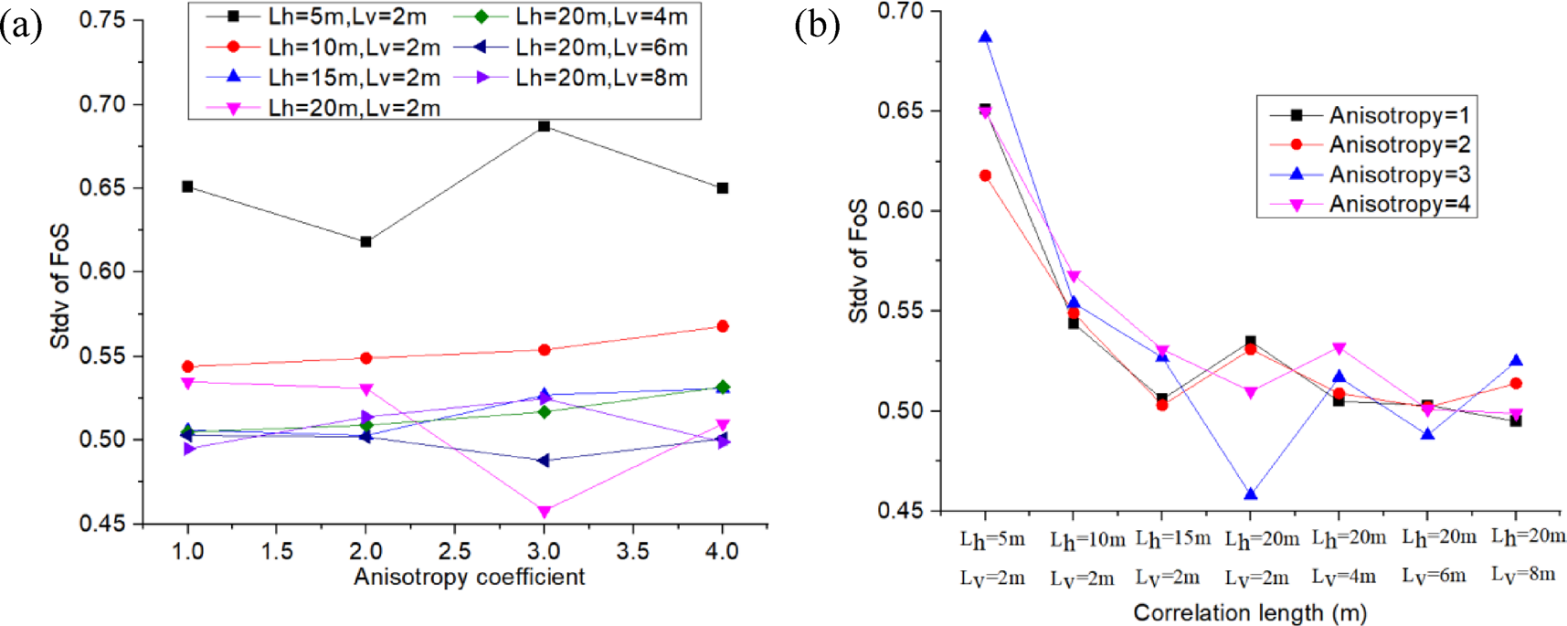
Influence of *ξ*, *L*_*h*_ and *L*_*v*_ on *σ*_*FoS*_.

**Fig. 19:**
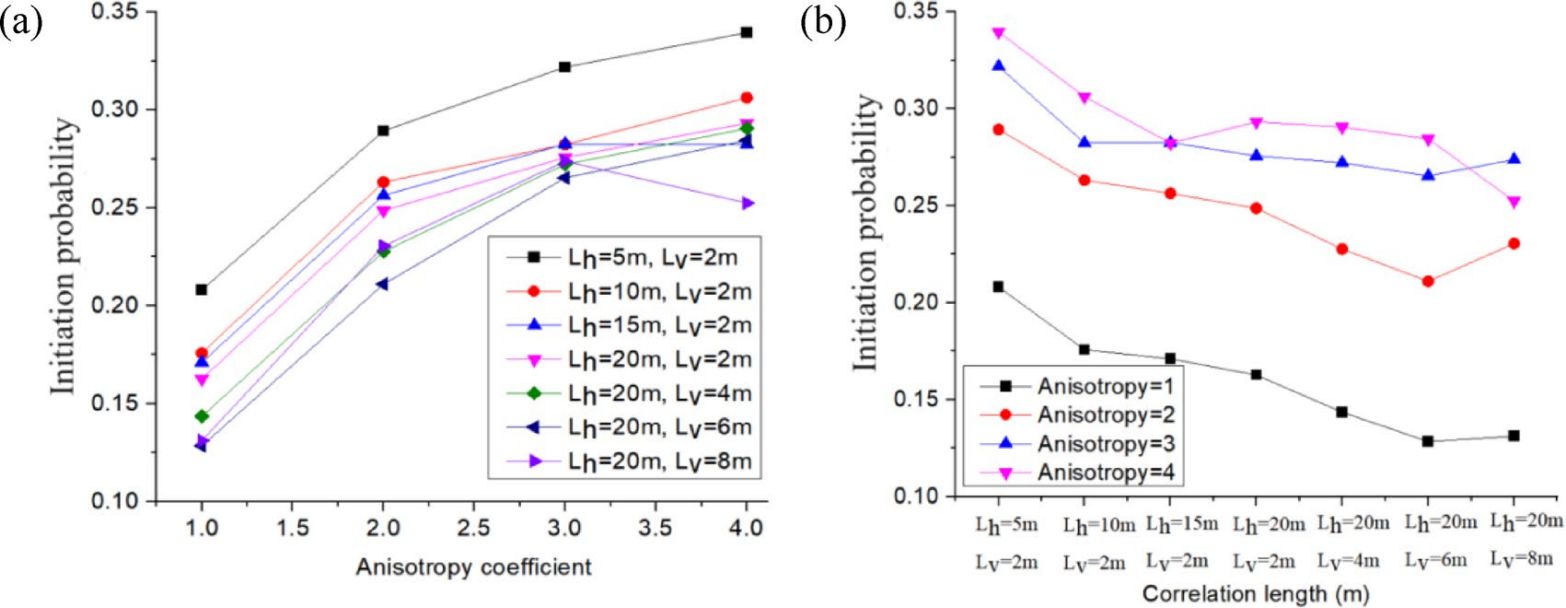
Influence of *ξ*, *L*_*h*_ and *L*_*v*_ on *P*_*f*_.

For this analysis, the earth dam was assumed to be under the same load condition (Upstream water level is 9.5 m), and the COV_Ks_ of the permeability RFs were kept constant at 0.75 for all subsequent cases. This section examines the anisotropy coefficient *ξ* and correlation lengths *L*_*h*_ & *L*_*v*_ of the permeability RFs to determine their impact on the estimated mean FoS (*µ*_*FoS*_), standard deviation of FoS (*σ*_*FoS*_), and initiation probability *P*_*f*_, which are obtained from the probabilistic seepage analysis (see Fig. 17, 18, 19). Table 5 shows the different values used for the anisotropy coefficient *ξ* and correlation lengths *L*_*h*_ & *L*_*v*_ in this sensitivity analysis.

**Table 5:** The anisotropy coefficient *ξ* and correlation lengths *L*_*h*_ & *L*_*v*_ influence on backward erosion.

Parameter	Value
Upstream water level *H* (m)	9.5
COV_Ks_	0.75
Anisotropy coefficient *ξ*	1, 2, 3, 4
Correlation length (m)	5 ≤ *L*_*h*_≤20, 2 ≤ *L*_*v*_≤8

Figure 17a shows that the estimated mean of the FoS (*µ*_*FoS*_) decreases significantly with an increase in the anisotropy coefficient *ξ*. Additionally, it shows that the rate of decrease of *µ*_*FoS*_ slows down as the anisotropy coefficient increases from 1 to 4. This is because increasing anisotropy leads to a higher horizontal permeability, which facilitates downstream flow and reduces FoS.

Figure 17b illustrates that *µ*_*FoS*_ may vary slightly with the correlation length, and the curves show the fluctuation of *µ*_*FoS*_. The first four points correspond to an increase in the horizontal correlation length *L*_*h*,_ and the last four points correspond to an increase in the vertical correlation length *L*_*v*_. Figure 17a,b show that the anisotropy coefficient has a stronger impact than the correlation length on the mean of FoS.

Figure 18a displays the standard deviation of FoS (*σ*_*FoS*_) with the variation in the anisotropy coefficient, and this is for different horizontal and vertical correlation length configurations. This figure shows that *σ*_*FoS*_ is not highly sensitive to the anisotropy coefficient *ξ.* The most significant fluctuations of the standard deviation *σ*_*FoS*_ as a function of the anisotropy coefficient are obtained for the configuration with a lower correlation length (e.g., the curve colored in black, *L*_*h*_= 5 m, *L*_*v*_ = 2 m). Figure 18b illustrates that *σ*_*FoS*_ decreases as the correlation length (and more particularly the horizontal correlation length *L*_*h*_) increases. This is because a higher correlation length can reduce the dispersion of the permeability random field (as shown in Fig. 7), which in turn decreases the standard deviation of permeability, hydraulic gradient, and FoS step by step. Conversely, using a configuration with a shorter correlation length will result in a greater dispersion of permeability. This, in turn, will increase the dispersion of the FoS at the base of the earth dam.

Figure 19a shows that the initiation probability *P*_*f*_ increases with the anisotropy coefficient, but the rate of increase can be slowed down with the anisotropy coefficient. As shown previously, the anisotropy coefficient has a minor effect on the standard deviation of the FoS (Fig. 18a), but a significant impact on the mean of the FoS (Fig. 17a). Therefore, an increase in the anisotropy coefficient results in a decrease in the mean FoS, which in turn increases the initiation probability due to backward erosion. Figure 19b shows that the initiation probability decreases overall with the correlation length. As observed previously, the correlation length has a minor effect on the mean of the FoS (Fig. 17b), but a significant impact on the standard deviation of the FoS (Fig. 18b). Therefore, an increase in the correlation length results in a decrease in the standard deviation of the FoS, which in turn reduces the initiation probability due to backward erosion.

## Discussion

This section presents a discussion on the of backward erosion initiation position and the assessment of the critical gradient using a local or global approach.

### Discussion on the backward erosion initiation location

Based on the results presented in Sect. 4, the potential initiation position for backward erosion (the node with the lowest FoS in the dam) varies across realisations in probabilistic seepage analyses based on Terzaghi criteria. In practical engineering, it is important to consider not only the initiation probability of backward erosion but also the initiation position. The lowest FoS value is only one of the necessary conditions for backward erosion. Another factor is the position of the node with the lowest FoS in the dam. Among all the realisations, the cases with the lowest FoS near the ground surface are more likely to initiate backward erosion. Therefore, this section highlights two configurations with different upstream water levels (e.g. *H* = 9 m & 9.5 m) to comprehensively evaluate the safety of the earth dam. What is more, the potential initiation position of backward erosion can be identified by locating the node with the lowest FoS in the dam.

**Fig. 20:**
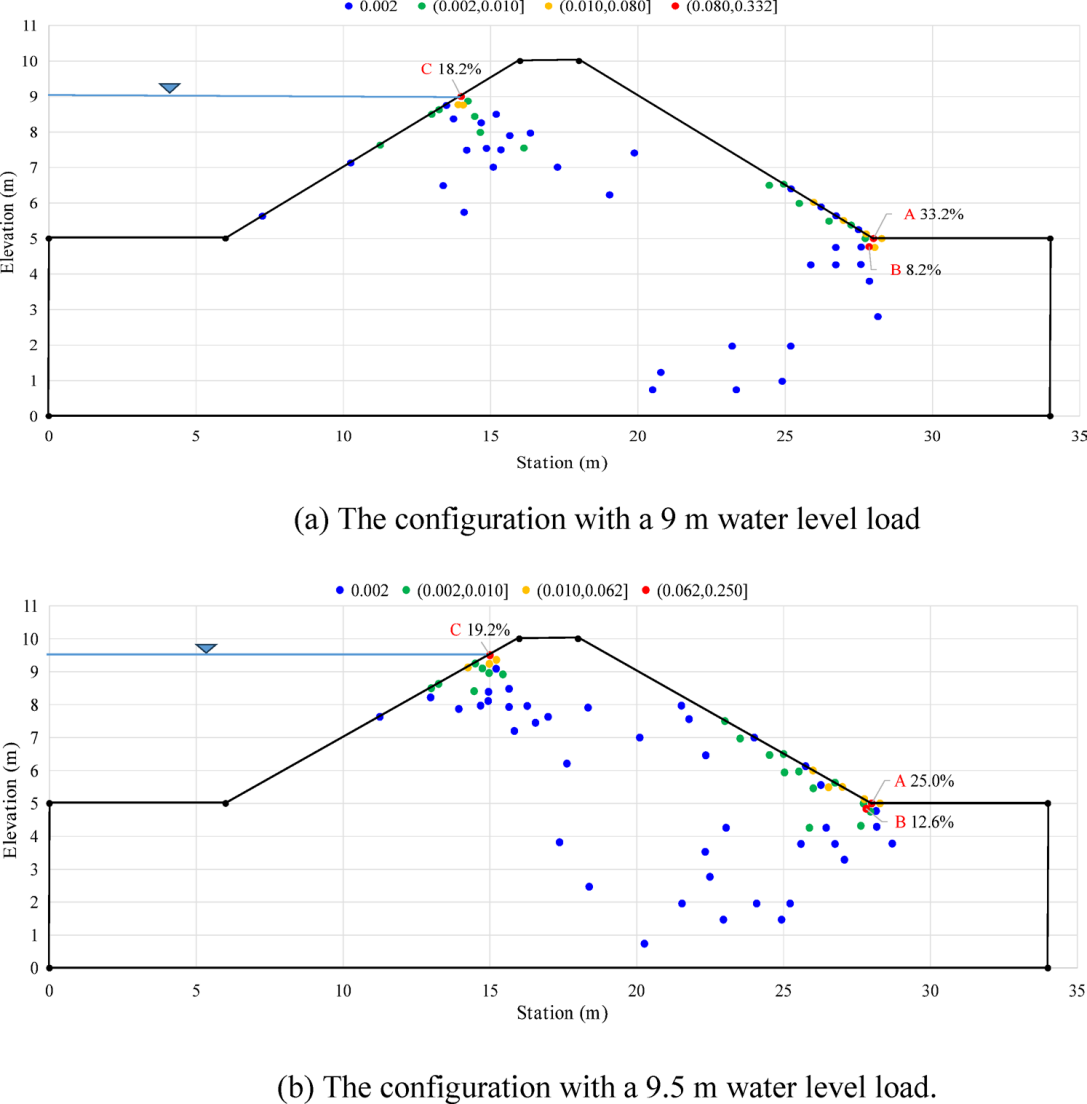
The distribution of the number of realisations (or probability) for the node with the lowest FoS (500 realisations in total).

The results of the 500 numerical simulations conducted in the probabilistic seepage analysis show that the lowest FoS is typically observed in the vicinity of the downstream toe (e.g. points A and B in Fig. 20) or near the surface of the reservoir level (e.g., points C in Fig. 20). However, the initiation of erosion is more critical around the downstream toe (exit gradient). To give an example, the lowest FoS was evaluated at point A for 166 cases out of the 500 realisations (33.2%) when the upstream water level is 9 m (see Fig. 20a).

Figure 20a,b illustrate the position of the points with the lowest FoS for each of the 500 realisations of the numerical simulations (considering the upstream water level at 9 m and 9.5 m in Fig. 20a,b, respectively). By observing Fig. 20a,b, it can be found that the potential initiation position (the point with the lowest FoS) is located more frequently at the downstream toe of the embankment, but the number of realisations in the configuration with a 9 m water level (166 realisations, 33.2%) is higher than that in the configuration with a 9.5 m water level (150 realisations, 25.0%). For the configuration with a water level of 9.5 m, there may be a lower dispersion of the FoS at the embankment toe, resulting in a lower value of *σ*_*FoS*_. This could explain the slight increase in *σ*_*FoS*_ when the water level increases from 9 m to 9.5 m (Fig. 14a).

Figure 20 shows that the point with the lowest FoS value in some cases can be located close to the surface of the reservoir level (e.g. point C in Fig. 20): in 18.2% of the cases when the water level is at 9.0 m and in 19.2% of the cases when the water level is at 9.5 m. However, the values of FoS at this node (point C) are higher than 1 in most of the realisations, which makes it difficult to initiate backward erosion from this point. Overall, when using the local approach for backward erosion analysis, it is necessary to calculate the FoS and *P*_*f*_ for each node of the dam and generate the corresponding distribution map as done in Sect. 4.1.1 (Fig. 11) in order to conduct a comprehensive safety assessment that includes the value of *P*_*f*_ and the initiation position.

### Comparision of the local approach and global approach

In addition to a local approach based on the Terzaghi model to evaluate the local critical hydraulic gradient at each point of the dam, practical engineering also considers simplified empirical models based on the evaluation of the global critical hydraulic gradient (such as the Sellmeijer and Hoffmans models). The aim of this section is to compare the results obtained using a local approach and a global approach to evaluate the safety of the dam against backward erosion (factor of safety FoS & initiation probability *P*_*f*_).

The criteria for backward erosion and the statistical properties of the soil parameters used in the Terzaghi, Sellmeijer and Hoffmans methods are given in Sect. 2.2, Tables 2 and 3. These elements are then used to compute the FoS and estimate the initiation probability *P*_*f*_ using Monte Carlo simulations (see Sect. 3.2).

The results of the FoS distribution (mean and standard deviation) and the initiation probability obtained for the Terzaghi method are presented in Sect. 4.1.1.

For the Sellmeijer and Hoffmans methods, the Monte Carlo simulations consisted of 500 random samples of the input data according to the distribution presented in Table 3 in order to evaluate the global critical hydraulic gradient of each sample and thus obtain a distribution of the global FoS. The results of the probabilistic backward erosion analysis (*µ*_*FoS*_, *σ*_*FoS*_, & *P*_*f*_) using the Terzaghi, Sellmeijer and Hoffmans methods are summarised in Table 6.

**Table 6:** The comparison of reliability analysis results using different backward erosion criteria.

Result parameters	Hoffmans method	Sellmeijer method	Terzaghi method
*µ*_*FoS*_	13.664	4.091	1.485
*σ*_*FoS*_	41.712	5.666	0.535
COV_FoS_	3.0527	1.385	0.360
*P*_*f*_	21.4%	22.2%	16.8%

The results presented in Table 6 show that the global approach (Sellmeijer method and Hoffmans method) may overestimate the initiation probability of backward erosion when compared to the local approach (Terzaghi method). The initiation probability for the Sellmeijer and Hoffmans methods is 21.4% and 22.2%, respectively, while for the Terzaghi method it is 16.8%. The Sellmeijer and Hoffmans methods give a high mean FoS, but with a high dispersion (standard deviation and COV), resulting in a higher initiation probability. As presented in Sect. 2.2, the Sellmeijer and Hoffmans models are based on a global critical hydraulic gradient approach, which takes into account several parameters associated with large uncertainties. This leads to a significant dispersion (e.g., COV) of the global critical hydraulic gradient and the global FoS, resulting in a higher estimated initiation probability in the Hoffmans and Sellmeijer models.

Although this study is based on a simplified dam geometry, it is important to note that backward erosion piping is a scale-sensitive process. Parameters such as hydraulic conductivity, particle size, and layer thickness may behave differently at full scale. Moreover, internal erosion is often a mechanism triggered locally, making it difficult to assess across the entire dam. These limitations are acknowledged, and future work will apply the method to real dam geometries and site-specific data to better evaluate scale effects.

## Conclusion

This study investigated a method for probabilistic backward erosion analysis that takes into account the spatial variability of seepage parameters in earth dams. The probabilistic seepage analysis is based on the random finite element method, which integrates random fields of soil hydraulic properties to obtain a random field of the hydraulic gradient. The method proposed allows obtaining, at any point of the dam, the distribution of the FoS and the initiation probability *P*_*f*_ of backward erosion. The stochastic seepage analysis of the dam against backward erosion included a sensitivity analysis to assess the influence of uncertainty on hydraulic parameters (e.g., reservoir water level, permeability anisotropy coefficient) as well as random field parameters (e.g., coefficient of variation of permeability and correlation lengths). In addition, two different criteria based on local hydraulic gradient (Terzaghi method) and global hydraulic gradient (Sellmeijer and Hoffmans methods) were used to calculate the initiation probability of backward erosion to compare the results between these approaches. Several conclusions can be drawn from this study:

1. The random finite element method proposed in this study can be used to assess the risk of backward erosion initiation in an earth dam, taking into account the spatial variability of soil properties. The method proposed makes it possible to obtain the FoS distribution (mean and standard deviation) and the initiation probability *P*_*f*_ of internal erosion initiation at each point of the dam. The resulting FoS and *P*_*f*_ distribution maps provide a clear and intuitive visualization of the most vulnerable zones, enabling the direct identification of potential backward erosion initiation areas within the dam body or foundation.
2. Parametric sensitivity analysis showed that the water level *H* and permeability anisotropy *ξ* have a strong influence on the mean factor of safety (FoS), while the coefficient of variation of permeability COV_Ks_ and the correlation lengths primarily affect its variability. Of all the parameters, *H*, *ξ*, and COV_Ks_ significantly impacted the backward erosion initiation probability *P*_*f*_, which increased with increasing values of these parameters, whereas the effect of correlation lengths was comparatively minor. This suggests that in practical applications, while careful characterization of anisotropy, permeability variability, and water level is essential, the selection of correlation lengths for random field generation can be approached with less stringency.
3. The initiation probability *P*_*f*_ of backward erosion was examined in this study using the local hydraulic gradient approach (Terzaghi method) and compared with results from global methods (Hoffmans and Sellmeijer). In the case study, the Sellmeijer and Hoffmans methods yielded higher mean FoS values, but also showed greater dispersion (standard deviation and COV), resulting in a higher *P*_*f*_. This suggests that, in practical applications, global methods may overestimate the probability of initiation due to compounded uncertainties, and local criteria may offer more stable and spatially resolved risk assessments when supported by detailed input data.

BEP is a progressive, time-dependent process involving detachment, transport, and enlargement phases. This study only focused on the initiation of backward erosion without considering the other phases of the erosion process relating to the progression of erosion until dam failure. Future work may incorporate erosion kinetics, transient flow, and coupled fluid–soil interaction models (e.g., CFD-DEM) to simulate the full backward erosion process.

Despite these limitations, the current framework provides practical insights by identifying zones where the probability of internal erosion initiation is higher. This information can aid engineers not only in prioritizing monitoring efforts, but also in evaluating the effectiveness of design measures—such as drainage systems, slope geometry, or filter configurations—to reduce the probability of backward erosion initiation in real dam projects.

## Data Availability

All associated data have been presented in the manuscript which is available from the corresponding author on reasonable request.
